# Single-cell nanobiopsy enables multigenerational longitudinal transcriptomics of cancer cells

**DOI:** 10.1126/sciadv.adl0515

**Published:** 2024-03-06

**Authors:** Fabio Marcuccio, Chalmers C. Chau, Georgette Tanner, Marilena Elpidorou, Martina A. Finetti, Shoaib Ajaib, Morag Taylor, Carolina Lascelles, Ian Carr, Iain Macaulay, Lucy F. Stead, Paolo Actis

**Affiliations:** ^1^ Faculty of Medicine, Imperial College London, London, UK.; ^2^ Bragg Centre for Materials Research, University of Leeds, Leeds, UK.; ^3^ School of Electronic and Electrical Engineering, University of Leeds, Leeds, UK.; ^4^ Leeds Institute of Medical Research at St James’s, University of Leeds, Leeds, UK.; ^5^ Earlham Institute, Norwich Research Park, Norwich, UK.

## Abstract

Single-cell RNA sequencing has revolutionized our understanding of cellular heterogeneity, but routine methods require cell lysis and fail to probe the dynamic trajectories responsible for cellular state transitions, which can only be inferred. Here, we present a nanobiopsy platform that enables the injection of exogenous molecules and multigenerational longitudinal cytoplasmic sampling from a single cell and its progeny. The technique is based on scanning ion conductance microscopy (SICM) and, as a proof of concept, was applied to longitudinally profile the transcriptome of single glioblastoma (GBM) brain tumor cells in vitro over 72 hours. The GBM cells were biopsied before and after exposure to chemotherapy and radiotherapy, and our results suggest that treatment either induces or selects for more transcriptionally stable cells. We envision the nanobiopsy will contribute to transforming standard single-cell transcriptomics from a static analysis into a dynamic assay.

## Teaser

Nanobiopsy enables the multigenerational, longitudinal sampling of the same cell (and its progeny) over 72 hours.

## INTRODUCTION

Microfluidics platforms coupled with advances in molecular biology and single-cell RNA sequencing (scRNA-seq) have enabled high-throughput analysis of the minute amount of genetic material contained in a single cell (*1*). The advent of spatial transcriptomics has then further enabled the generation of single-cell gene expression data while maintaining the spatial context of those cells within a tissue (*2*). However, all these approaches are end-point assays that require the cell to be lysed or fixed before profiling. An exciting frontier in single-cell analysis is the facilitation of temporal/sequential/longitudinal cellular profiling (*3*, *4*).

Gene expression is intrinsically dynamic. Cells in a heterogeneous system exist in different transcriptional states and can transition from one state to another over time and in response to stimuli (*5*, *6*). Measuring gene expression over time enables the determination of cellular trajectories, which refer to the sequential gene expression patterns that cells follow as they transition between transcriptional states. The determination of these trajectories is fundamental to unraveling the complex regulatory networks and molecular mechanisms responsible for developmental processes, disease progression, cellular adaptation, and overall cellular functioning (*7*, *8*).

Several techniques have been developed in the past years to enable the time-series analysis of gene expression in individual cells, and they rely on computational methods based on statistical inference combined with experimental molecular approaches such as metabolic labeling, cell type–specific reporters, and genetic barcodes (*7*, *9*). However, these experimental methods have the limitation of operating within short time frames, while computational approaches rely on specific biological assumptions to infer the cell’s initial state (*10*). Alternative approaches in nanotechnology have been developed to overcome these limitations.

Guillaume-Gentil *et al.* (*11*) and Chen *et al*. (*12*) established Live-seq, a technique based on fluidic force microscopy allowing the pressure-driven RNA extraction from living cells at distinct time points. The authors sequentially profiled the transcriptomes of individual macrophages before and after lipopolysaccharide stimulation and of adipose stromal cells pre- and postdifferentiation (*12*). Nadappuram *et al.* (*13*) developed minimally invasive dielectrophoretic nanotweezers based on dual carbon nanoelectrodes that allowed the trapping and extraction of nucleic acids and organelles from living cells without affecting their viability. The authors demonstrated the single organelles resolution of their techniques but did not demonstrate longitudinal sampling. Alternative approaches relying on vertical nanoneedle platforms have the potential to enable high throughput single-cell manipulation (*14*) and they recently enabled the intracellular sampling from a few cells to investigate cellular heterogeneity and temporal dynamics of microRNA expression (*15*).

In parallel with the progress in gene expression profiling, the emergence of gene editing and immunotherapy has driven the creation of innovative approaches for delivering exogenous molecules into individual cells. Mukherjee *et al.* (*16*) introduced an automated electroporation system designed for delivering plasmids, proteins, and Cas9-ribonucleoprotein complexes for gene editing of hard-to-transfect cells. In addition, vertical nanoprobes have been established as effective tools for intracellular delivery of exogenous molecules (*14*, *17*). Their use offers notable advantages in enhancing the transfection efficiency of immune cells, which is crucial, for example, in generating chimeric antigen receptor (CAR)–T cells for immunotherapy (*18*–*20*).

In essence, the capability of these versatile nanoprobes to access the intracellular environment with minimal disruption holds the potential to revolutionize molecular diagnostics, gene, and cell therapies. This potential has been realized in recent years through various industrial and clinical applications, with further growth anticipated in the future (*21*).

Our group has pioneered the use of nanopipettes coupled with electrowetting and integrated into a scanning ion conductance microscope (SICM) (*22*, *23*) to perform nanobiopsies of living cells in culture (*24*). SICM relies on the measurement of the ion current between an electrode inserted in a glass nanopipette, the probe, and a reference electrode immersed in an electrolytic solution where the cells are placed (*23*). By applying a voltage between the two electrodes, an ion current flows through the nanopore at the tip of the nanopipette. When the nanopipette approaches a surface, the measured ion current drops. This current drop is proportional to the separation between the nanopore and the sample and can be used as active feedback to maintain the nanopipette-sample distance constant, and it has been extensively used for high-resolution topographical mapping of living cells (*23*). The nanobiopsy technique has enabled the extraction of RNA and organelles from single cells, the study of mRNA compartmentalization within neuronal cells (*25*), and the localized sampling of mitochondria from human tissues (*26*).

Here, we show the development of a platform technology enabling the extraction of femtoliter volumes of cytoplasm and the simultaneous injection of exogenous molecules into individual cells, via a double-barrel nanopipette. This platform offers several advantages compared to previously established techniques. First, the high conductivity of the aqueous barrel greatly facilitates nanopipette operations, enabling automated positioning through feedback control. Moreover, the monitoring of ion current signals allows for the detection of current signatures, serving as an indicator of successful membrane penetration, as confirmed by the injection of a fluorophore, ensuring the nanobiopsy takes place within the cell cytoplasm. Last, the capability to introduce exogenous molecules before nanobiopsy considerably broadens the scope of applications for the technique, as it can be integrated with cutting-edge methodologies, including molecular labeling and gene editing.

The platform enables multigenerational longitudinal nanobiopsy of the same cell (and its progeny) to profile gene expression changes over 72 hours. As a proof of concept, we applied our novel method to investigate changes in gene expression in a model of the most aggressive brain cancer, glioblastoma (GBM), caused by nonsurgical elements of the standardized treatment given to patients: radiotherapy and chemotherapy with the drug temozolomide (TMZ).

GBM is a fatal brain cancer. All tumors recur after treatment, constituting a major unmet clinical need. Intratumor heterogeneity is common in GBM with numerous cells existing in different states within the same tumor. The transition of cells between different states confers plasticity to the tumor and it is believed to be responsible for cellular adaptation through therapy (*27*–*29*). While previous single-cell studies have detected changes in GBM cell phenotype distribution following treatment, they are unable to determine whether this shift is due to alterations of cell division and death rates across different cell types, changes in cell progeny phenotypes, or instead due to direct switching from one cell phenotype to another. Our technology allowed for the sampling of the same cell and its progeny longitudinally, through therapy, and enabled the determination of phenotype changes that may underpin the ability of these cells to adapt and resist treatment.

## RESULTS

**Fig. 1.**
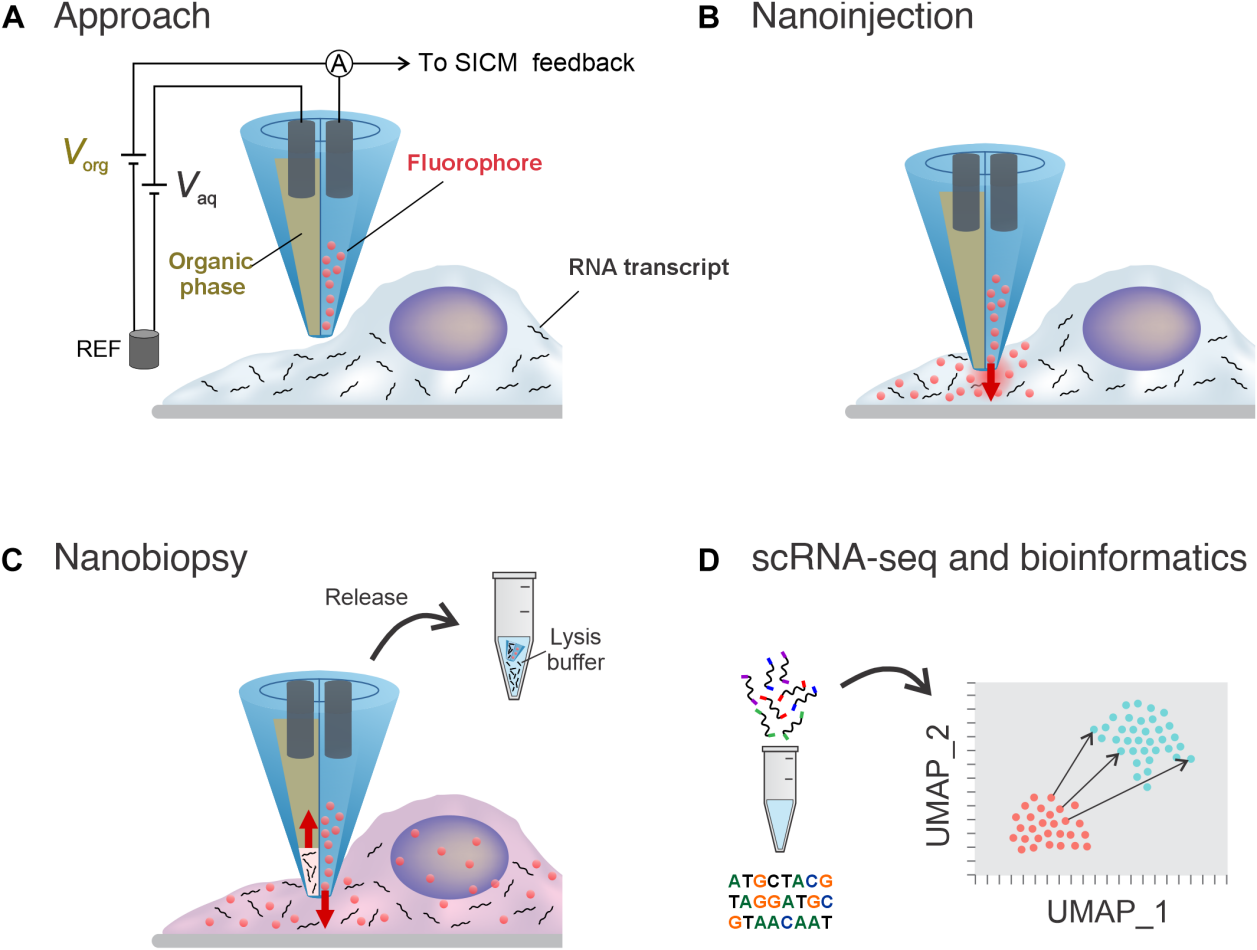
Illustration of the main phases of the single-cell nanobiopsy procedure. A double-barrel nanopipette integrated into a SICM is brought in proximity of the cellular membrane (**A**) where it penetrates the latter enabling the release of the fluorophore through the aqueous barrel (**B**) and cytoplasmic extractions through the organic barrel (**C**). After the extraction, the sample containing RNA transcripts is released in a lysis buffer and it is reverse-transcribed, amplified, and sequenced for downstream gene expression analysis via bioinformatics (**D**).

Double-barrel nanopipettes integrated into a SICM enable the injection into living cells followed by extraction of a minute amount of the cytoplasm for downstream gene expression profiling. Our platform technology uses a double-barrel nanopipette (individual barrel with a pore of ~150 nm; fig. S1) where one barrel, filled with an organic solution, is used as an electrochemical syringe to perform cytoplasmic extractions (*24*–*26*, *30*, *31*), while the second barrel, filled with an aqueous electrolyte solution, provides a stable ion current for precise positioning and nanoinjection of exogenous molecules into the cell. These are distinct advantages to the work by Nashimoto *et al.* (*31*) where double-barrel nanopipettes were only used for improved positioning before nanosampling and from Seger *et al.* (*32*) who used double-barrel nanopipettes to perform intracellular injections at high voltages (>10 V). Also, Saha-Shah *et al.* (*33*) used double-barrel nanopipettes as push-pull probes to sample from *Allium cepa* cells but did not demonstrate sequential injection and extraction. Figure 1 illustrates the nanobiopsy technology and its workflow composed of automated nanopipette approach to the cell (Fig. 1A), penetration of the cell membrane followed by nanoinjection (Fig. 1B), extraction of cytoplasmic RNA via nanobiopsy (Fig. 1C), and transcriptomics analysis based on next-generation sequencing and bioinformatics (Fig. 1D).

### Approach

In the approach phase, the double-barrel nanopipette automatically approaches the cell membrane under the control of the SICM interface operating in hopping mode (*34*). The electrolyte solution in the aqueous barrel is characterized by an electrical conductivity κ_aq_ = 1.30 S/m which is two orders of magnitude higher than the conductivity of the organic solution κ_org_ = 0.01 S/m (*30*). This results in a median electrical resistance of the aqueous barrel *R*_aq_ = 45.7 megohms being 59 times smaller than the median resistance of the organic barrel *R*_org_ = 2.7 gigohms which translates with higher values of ion current magnitude that facilitate the SICM operations. A positive bias is applied to the electrode in the aqueous barrel (*V*_aq_ = 200 mV) with respect to a reference electrode immersed in the culture medium. When the nanopipette approaches the plasma membrane, the magnitude of the ion current decreases due to the increase in the nanopipette access resistance (*22*, *35*) until the set point current (99.5% of the baseline current magnitude) is detected.

At this point, the SICM feedback mechanism stops and retracts the nanopipette to keep a 2-μm distance from the cell membrane. The ion current through the aqueous barrel *i*_aq_ corresponding to the vertical position *z* of the nanopipette recorded during the approach phase, the boxplots showing the resistance of the organic and aqueous barrel, and the optical micrographs of the process are shown in figs. S2 and S3. For the entire duration (approximately 1 min) of the approach phase, the potential applied to the electrode in the organic phase is kept at a constant positive value (*V*_org_ = 300 mV) to prevent the aqueous phase from entering the nanopipette (*24*, *30*).

### Nanoinjection

In the nanoinjection phase, the nanopipette position is manually controlled to enable the reproducible penetration of the cell membrane. Following the approach (subphase 1: hopping), the nanopipette is lowered by 2 μm to reach the cell membrane level, then the potential applied to the aqueous barrel is switched to *V*_aq_ = −500 mV to allow the electrophoretic release of the anionic fluorophore from the nanopipette. Next, the nanopipette is lowered at high speed (10 nm/ms) with 100-nm steps until we detect a ~2% drop in the ion current magnitude that we assign to the nanopipette touching the cell membrane (subphase 2: membrane touch). The nanopipette is then further lowered until we detect a vibrational noise, which we attribute to the proximity of the nanopipette tip to the petri dish (subphase 3: vibrational noise). A further 100-nm step brings the nanopipette in contact with the polymer coverslip of the petri dish and no ion current is detected (*i*_aq_ = 0 pA). Last, the nanopipette is retracted by 100 nm and it is kept at a fixed position for approximately 1 min to allow the release of a sufficient amount of fluorophore into the cytoplasm.

**Fig. 2.**
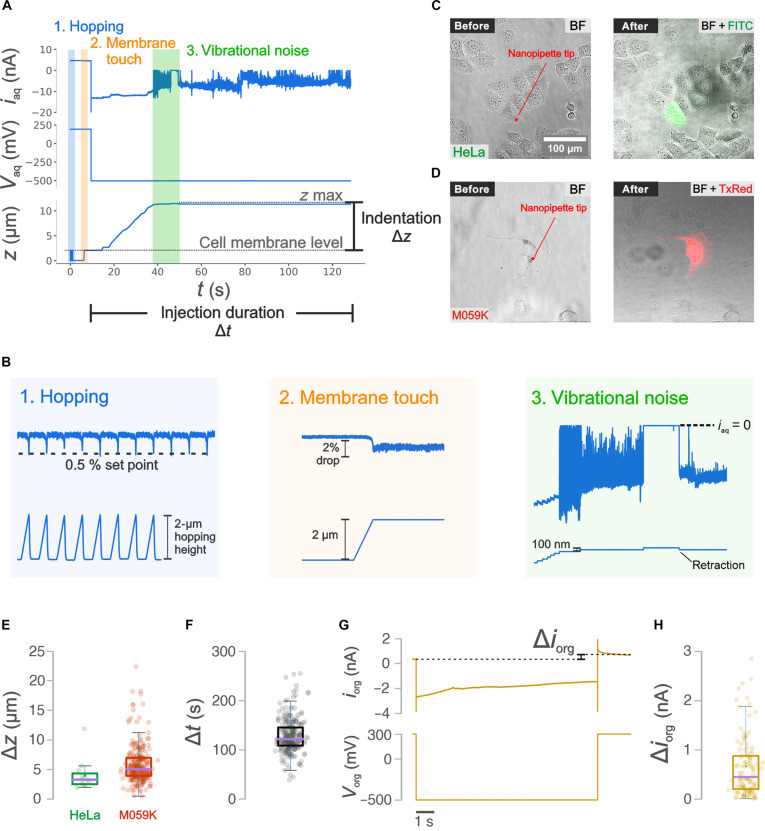
Electrical characterization of the nanoinjection and nanobiopsy process. (**A**) Electrical traces for the ion current *i*_aq_ and potential bias *V*_aq_ in the aqueous barrel and *z*-position of the nanopipette *z* during the nanoinjection phase where the shaded regions highlight the hopping (1), membrane touch (2), and vibrational noise (3) subphases. (**B**) Zoomed-in traces for the ion current *i*_aq_ (top) and nanopipette vertical position *z* (bottom) recorded during the hopping (1), membrane touch (2), and vibrational noise (3) subphases whose detection guarantees a successful membrane penetration and nanoinjection. (**C**) The bright-field (BF) and fluorescence [filter set for Fluorescein isothiocyanate (FITC) and Texas red (TxRed)] micrographs of a HeLa and M059K (**D**) cell acquired before and after the nanoinjection phase show the cell emitting a fluorescent signal following injection of a green (ATTO 488) and red (ATTO 565) fluorophore, respectively. (**E**) Boxplots showing the maximum indentation Δ*z* in the case of nanoinjections performed into HeLa (*n* = 11) and M059K cells (*n* = 256) and (**F**) the total injection duration Δ*t* (*n* = 256). (**G**) Traces of the ion current *i*_org_ and applied voltage *V*_org_ in the organic barrel during nanobiopsy. After the extraction (*t* > 10 s), the ion current *i*_org_ is characterized by a higher magnitude due to the cytoplasm entering the nanopipette which decreases the nanopipette resistance. (**H**) Boxplot showing the distribution of the current magnitude increase Δ*i*_org_ (*n* = 117).

Figure 2A shows the ion current *i*_aq_ and the potential applied *V*_aq_ in the aqueous barrel and the vertical position *z* of the nanopipette during nanoinjection, while the zoomed-in traces for the individual approach (1), membrane touch (2), and vibrational noise (3) phases are shown in Fig. 2B. Note that the nanopipette vertical position *z* increases as the distance between nanopipette and cell membrane decreases. The power spectral density of the ion current *i*_aq_ trace can be used as a quality control measure, as the detection of the individual subphases strongly correlated with a successful nanoinjection, resulting in a fluorescently labeled cell (sections S1.4 and S1.5 and figs. S4 to S6). Figure 2 (C and D) shows the optical micrographs obtained before and after the nanoinjection of a green anionic fluorophore (ATTO 488) into a HeLa cell and a red anionic fluorophore (ATTO 565) into an M059K cell. Figure 2E shows the indentation of the nanopipette onto the cellular membrane necessary to puncture the membrane and access the cytoplasm extracted from the traces shown in Fig. 2A. The boxplots show a median value (purple line) of Δ*z* = 3.32 μm and Δ*z* = 5.06 μm and an average (green triangle) of Δ*z* = 4.12 μm and Δ*z* = 5.85 μm in the case of HeLa and M059K cells, respectively. The distribution for total injection duration Δ*t* is shown in the boxplot in Fig. 2F where the median value is Δ*t* = 129 s.

The mechanism involved in the cell membrane penetration is primarily mechanical, as the applied potential alone is insufficient to cause membrane rupture unless a gigaseal is established between the nanopipette and the cellular membrane. Cancer cells may exhibit greater stiffness than healthy cells, and stiffness changes among different cell lines (*36*, *37*). Moreover, sharper nanopipettes provide a higher localized force which results in lower membrane deformation, but this comes at the expense of a considerably reduced extracted volume in the nanobiopsy process. Our method is robust and reproducible, allowing membrane penetration and nanoinjection across different cell types (HeLa: epithelial cell; M059K: glial cell) with distinct mechanical properties. The average success rate of nanoinjection is equal to 0.89 ± 0.07, as illustrated in fig. S7.

### Nanobiopsy

Following the nanoinjection phase, our platform technology enables the extraction of intracellular RNA. Figure 2G shows an example of the ion current *i*_org_ and the potential *V*_org_ applied to the electrode in the organic barrel during the nanobiopsy phase where the electric potential is switched to *V*_org_ = −500 mV for 10 s. Upon switching the potential, the magnitude of the ion current in the organic barrel increases from *i*_org_ = 0.35 nA to *i*_org_ = −2.83 nA and it decreases slowly until the end of the 10 s when the potential is returned to *V*_org_ = 300 mV. This behavior is driven by the velocity of the displacement of the liquid-liquid interface between the solution in the barrel (organic phase) and the cytoplasm (aqueous phase) which is fast at the beginning and decreases over time. At this point, the magnitude of the ion current is approximately equal to *i*_org_ ~ 2.20 nA and it slowly decreases until reaching *i*_org_ = 0.70 nA, when the equilibrium is re-established. In the example trace, the increase in current magnitude due to ingress of cytoplasm after the extraction is equal to Δ*i*_org_ = 0.35 nA. Figure 2H shows the boxplot with the distribution of the current magnitude increase Δ*i*_org_ obtained after the extraction phase of 117 nanobiopsied cells with a median value (purple line) equal to Δ*i*_org_ = 0.40 nA. The value *i*_org_ following nanobiopsy can be used to calculate the nanopipette resistance and estimate the cytoplasmic volume extracted for a nanopipette geometry approximated to a truncated hollow cone (*30*). Our estimation indicates that 75% of samples show an extracted volume of ≤200 fl. However, this figure is only qualitative and cannot be used to precisely estimate the extracted volume. Nevertheless, Fig. 2H demonstrates the reproducibility of the protocol and indicates that it is very likely that less than a picoliter is extracted from the cell. Further ion current traces recorded during nanobiopsy are shown in fig. S8.

### Single-cell RNA sequencing

Following nanobiopsy, the nanopipette is placed in a tube preloaded with 2 μl of lysis buffer containing a ribonuclease (RNAse) inhibitor to prevent mRNA degradation and immediately immersed in liquid nitrogen for short-term storage. The time elapsed from the nanopipette withdrawal to the storage of the sample in liquid nitrogen was <3 min and includes the time required to withdraw the nanopipette from the culture solution, break the nanopipette tip in the tube containing lysis buffer, and immerse it in liquid nitrogen. Next, the mRNA collected is reverse-transcribed into cDNA and amplified according to Smart-Seq2 (*38*), and single-cell libraries are prepared for subsequent next-generation RNA sequencing and gene expression profiling.

### Longitudinal nanobiopsies of individual glioblastoma cells

**Fig. 3.**
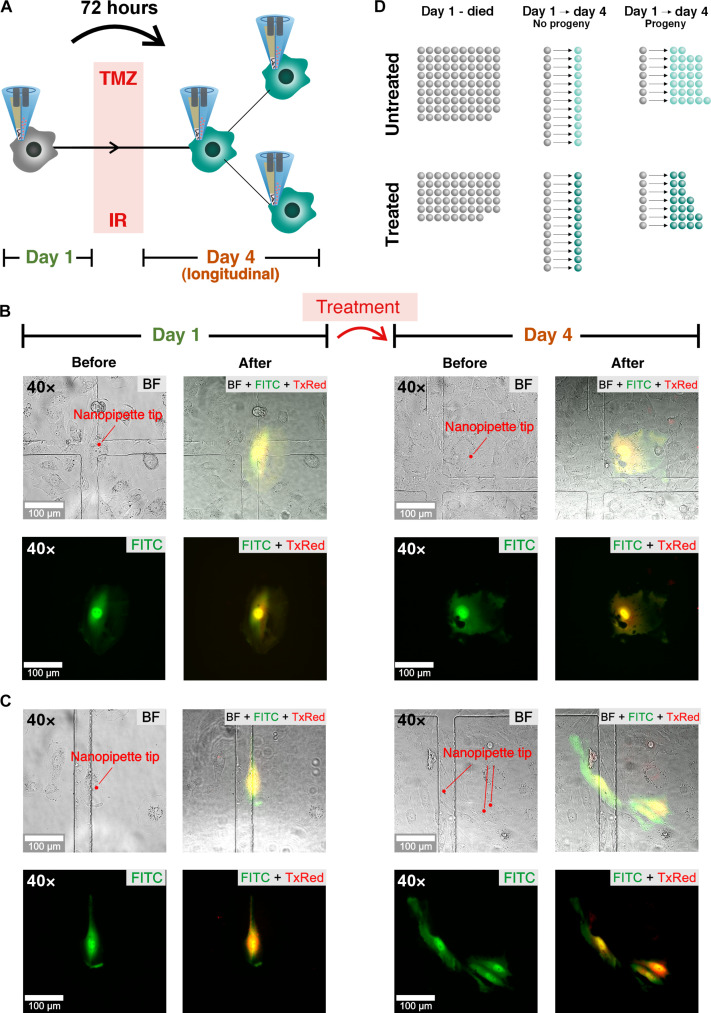
Longitudinal nanobiopsy and nanoinjection of individual glioblastoma cells through therapy. (**A**) An individual GBM cell undergoes nanobiopsy and nanoinjection on day 1. Following standard treatment of temozolomide TMZ and irradiation (IR) the same cell or its progeny undergoes a second nanobiopsy and nanoinjection on day 4 (longitudinal), 72 hours after the first nanobiopsy. (**B**) Optical (BF) and fluorescence (FITC, TxRed) micrographs of an individual M059K_GFP_ cell before and after the first (day 1) and longitudinal nanobiopsy following standard treatment (day 4). (**C**) Optical and fluorescence micrographs of an individual M059K_GFP_ cell undergoing initial nanobiopsy and nanoinjection (day 1), surviving treatment and dividing, and subsequent longitudinal nanobiopsy and nanoinjection of its progeny following treatment (day 4). (**D**) Illustration of the nanobiopsy count of untreated and treated cells that were nanobiopsied on day 1 and died (day 1–died), nanobiopsied on day 1, survived, did not divide, and nanobiopsied again on day 4 (day 1 and day 4–no progeny) and cells that were nanobiopsied on day 1, divided, and whose progeny was nanobiopsied again on day 4 (day 1 and day 4–progeny).

The nanobiopsy technology allows the longitudinal gene expression profiling of single cells in culture. As a proof of concept, we investigated the transcriptional response of GBM cells to physiologically relevant doses of the treatment received by patients: 2-gray (Gy) radiation and 30 μM TMZ chemotherapy. M059K GBM cells are amenable to nanobiopsy and can be visually tracked, along with their progeny, over a 72-hour time frame without leaving the field of view. Topographical mapping with SICM and fluorescent staining was performed on some of the M059K GBM cells to assess the average cell height, surface area, and volume; this information was then used to guide the location of the nanobiopsy procedure (figs. S9 and S10). For consistency, all nanobiopsies were carried out in the perinuclear region, and fluorescent staining indicated the presence of mitochondria and the endoplasmic reticulum in that region (fig. S10). To enable longitudinal tracking of individual GBM cells over time, mixed cultures of green fluorescent protein (GFP)–transfected (M059K_GFP_) and wild-type (M059K_WT_) cells were plated on gridded dishes at a ratio of 1:350 (M059K_GFP_:M059K_WT_). This ratio facilitated the identification of individual M059K_GFP_ surrounded by M059K_WT_, and it was crucial to ensure that after 72 hours, we could distinguish the original M059K_GFP_ cell or its progeny from any other M059K_GFP_ cells present in the dish at that time. The experimental setup and the use of gridded dishes to locate cells across multiple generations are outlined in the supporting information (section S2.3). To perform longitudinal analysis, it is crucial to ensure that the same cell or its progeny is sampled. Therefore, the total (Δ*x*_tot_) and maximum (Δ*x*_max_) cellular migration over the planned 72-hour time course was quantified and showed not to differ between repeats (*t* test, *P* > 0.5) and to be substantially less (Δ*x*_max_ < 800 μm, Δ*x*_max_ < 600 μm) than the field of view (1300 μm × 1300 μm). Furthermore, the largest area enclosing all progeny, in cases where the cell divided over the time course, was 0.35 mm^2^ which is ~5 times smaller than the area of the field of view (1.69 mm^2^) (fig. S11). It is crucial to assess the likelihood of obtaining a longitudinal biopsy from the same cell by factoring in variables such as migration speed, division rate, and progeny death rate. Our estimation suggests that the chance of a cell migrating beyond the field of view within this timeframe is equal to 2% (section S2.4 and fig. S12). Consequently, if the cell survives without dividing, then we can confidently confirm that the same cell was biopsied again after 72 hours. However, if the cell undergoes division, creating progeny, then the probability of identifying the same cell in the biopsy after 72 hours depends on the number of resulting progeny cells and their likelihood of survival. In our analysis involving three divisions (resulting in eight cells present at the same location after 72 hours), we estimated this probability to be approximately 14%. Note that our method cannot definitively distinguish whether the biopsied cell is the same as the initial cell or one of its progeny. Nonetheless, we can assert with confidence that either the same cell or its progeny was biopsied after 72 hours. Next, we tested whether the same M059K GBM cell could be sampled at different time points, and we followed the same cell over the 72-hour time course. Results showed that the cell was successfully biopsied and injected on day 1, imaged on days 2 and 3, and longitudinally biopsied and injected on day 4 (fig. S13). Having established the suitability of the nanobiopsy for a longitudinal sampling of the same GBM cell, we performed a longitudinal sampling of M059K GBM cells through standard therapy as depicted in Fig. 3A. Single M059K_GFP_ cells were identified, and their location was recorded. A first nanobiopsy of the cell was executed to extract cytoplasm (day-1 sample) and inject a fluorescent dye. This was repeated on day 4 on the same cell or each progeny thereof. On day 2, half of the dishes had been subjected to nonsurgical elements of standard GBM treatment, including 2-Gy irradiation (IR) and 30 μM TMZ, while the remaining half were left untreated. Figure 3B shows the optical micrographs of an individual M059K_GFP_ cell that was biopsied and injected on day 1, treated, and longitudinally biopsied on day 4, while Fig. 3C shows the case of an individual M059K_GFP_ that was biopsied and injected on day 1 and that divided following standard therapy, and whose progeny was longitudinally biopsied and injected on day 4. Similar examples for the longitudinal samples collected from the untreated cells are shown in fig. S14. The total number of nanobiopsies taken for each group (treated and untreated) at each time point (days 1 and 4) is summarized in the schematics in Fig. 3D. A total of 256 samples were collected from M059K_GFP_ cells of which 71 were longitudinal samples from 32 treated and 39 untreated cells. Twelve treated and 12 untreated cells sampled longitudinally did not divide over the 72 hours, while 7 untreated and 7 treated cells sampled longitudinally divided.

**Fig. 4.**
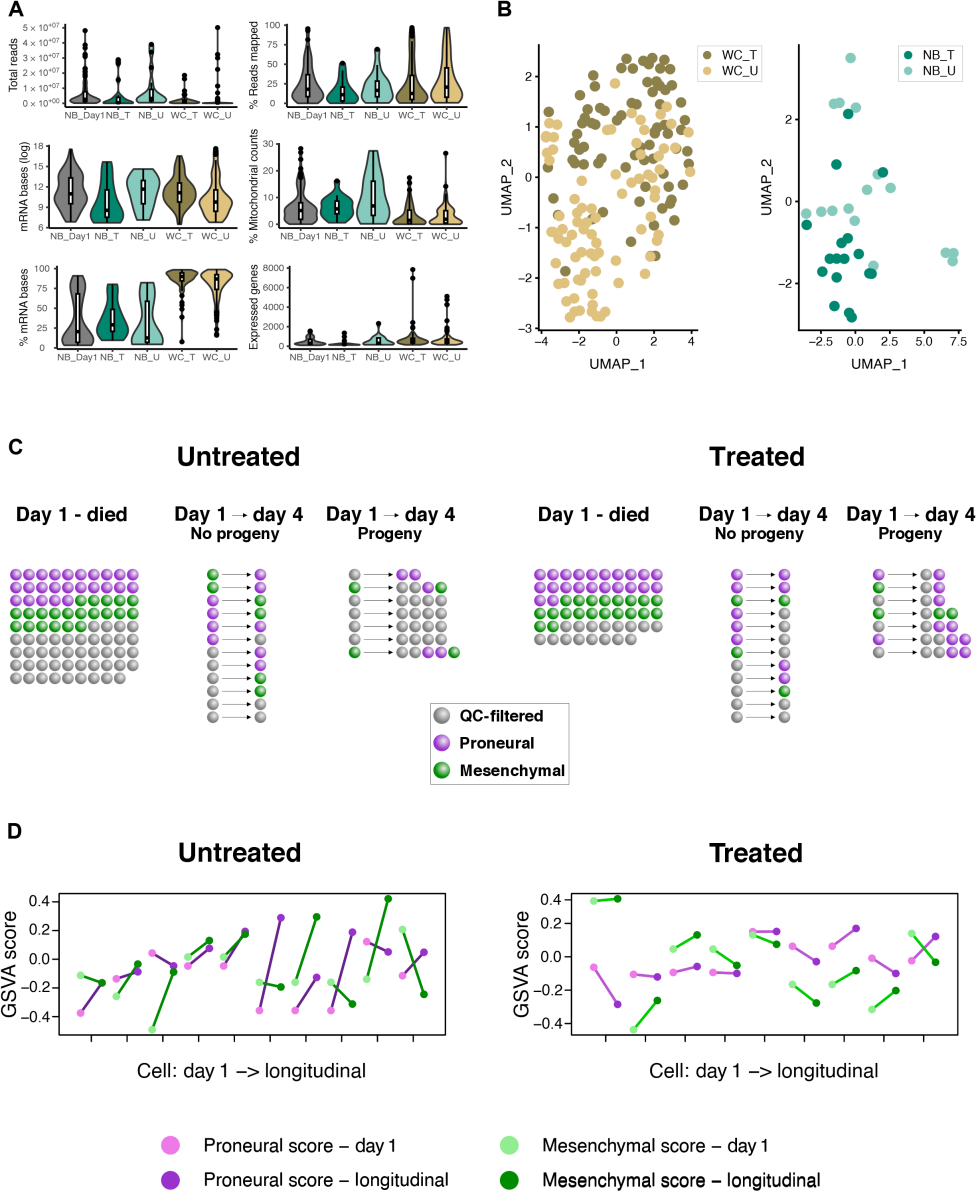
Gene expression profiling of longitudinal nanobiopsy samples determines the phenotype changes of individual glioblastoma cells through therapy. (**A**) Main sequencing metrics of day-1 nanobiopsy (NB_Day1), longitudinal nanobiopsy of treated (NB_T) and untreated (NB_U), and whole-cell lysate treated (WC_T) and untreated (WC_U) samples. (**B**) Uniform Manifold Approximation and Projection (UMAP) visualization of treated and untreated whole-cell lysate and longitudinal nanobiopsy samples. (**C**) Nanobiopsy count after quality control (QC) filtering (gray), and gene set variation analysis (GSVA) scoring for the proneural (purple) and mesenchymal (green) phenotypes. (**D**) Cell scores from GSVA of proneural (purple) and mesenchymal (green) phenotypes for the paired day-1 (light color) and longitudinal (dark color) samples. The crossing of the lines indicates a subtype switch of the cell.

When comparing treated and untreated cells, there was no significant difference in the number of cells that underwent day-1 nanobiopsies and died versus survived (chi-square, *P* = 0.24) nor that survived and divided versus survived and did not divide (chi-square, *P* = 1.0). The impact of nanobiopsy on cell viability was assessed by comparing the survival rate of cells over 72 hours following nanobiopsy with that of the control cells, which were plated and monitored for the same duration. Among the untreated biopsied cells, 19 out of 108 (~18%) survived, whereas 19 out of 77 (~25%) treated biopsied cells survived over 72 hours. In contrast, we observed a higher survival rate of 26 out of 41 (~63%) for the control cells (not biopsied, untreated) during the same 72-hour duration (fig. S15). We evaluated the effects of nanobiopsy on cell division by comparing the cell population 72 hours after nanobiopsy to the control group. Results suggest a reduced division rate in the nanobiopsied cells (fig. S16). We then assessed the effect of nanobiopsy on cell mobility by calculating the maximum cell migration over 72 hours after biopsy using the methodology described in the Supplementary Materials (section S2.3) and comparing it to the control group. Results indicated a reduced mobility for cells that were treated and did not generate progeny over the 72-hour post-biopsy when compared to the control group (fig. S17). Nevertheless, as this reduced mobility was not observed in the case of other biopsied cells, it suggests that this difference may not be directly attributable to the biopsy procedure itself but, potentially, to the effects of senescence induced by ionizing radiation (*39*, *40*). Sequencing libraries were created using SmartSeq2 from the extracted cytoplasm samples, as well as from whole-cell lysates of treated and untreated M059K as a comparison (fig. S18). Quality metrics of the two datasets (fig. S19) were generally comparable, though the nanobiopsies had higher total reads (mean = 6.5 × 10^6^ versus 2.8 × 10^6^), fewer expressed genes (mean = 497 versus 1100), lower % of bases aligning to mRNA regions (mean = 28 versus 82), and higher % mitochondrial gene counts (mean = 23.4 versus 7.5). Both datasets were filtered for >150 expressed protein-coding genes, <10% ribosomal bases, and <30% mitochondrial gene counts, with the latter allowing for the high concentration of mitochondria in the nanobiopsied region. The sequencing metrics after filtering are shown in Fig. 4A, with 159 of 192 (82.8%) whole cells and 145 of 274 (52.9% including 5 replicates) biopsies passing the criteria [*N* = whole-cell untreated (WC_U): 82, whole-cell treated (WC_T): 77, nanobiopsy day 1 (NB_Day1): 107, nanobiopsy longitudinal untreated (NB_U): 16, nanobiopsy longitudinal treated (NB_T): 17]. While a lower % of the total nanobiopsy bases were mRNA compared with whole-cell bases (Fig. 4A, bottom left), the much higher read depth for nanobiopsies meant that a comparable number of mRNA bases were captured for both datasets (Fig. 4A, middle left) for use in downstream analysis. The impact of the different volume amounts extracted on the sequencing data was evaluated by calculating the correlation between the increase in the ion current magnitude Δ*i*_org_ and the quality metrics. Results suggest a slight correlation between Δ*i*_org_ and the total number of reads (Spearman’s coefficient = 0.23) and the number of expressed genes (Spearman’s coefficient = 0.16), but the extent of the correlation introduces only minimal bias due to different amounts of extracted volume (fig. S20). Moreover, the contamination caused by the culture medium and the effect of the electrowetting on the amount of extracted mRNA were evaluated by comparing the sequencing metrics of the nanobiopsy samples to the sequencing metrics of the samples collected by immersing the nanopipette in the medium with the cultured cells without applying electrowetting (media control) and the samples collected by penetrating the cellular membrane of a cell without applying electrowetting (inside cell control). The sequencing metrics indicate that the number of expressed genes and the percentage of mRNA bases are significantly higher in the case of the nanobiopsy samples when compared to the control groups (fig. S21). Gene expression profiles were generated for each nanobiopsy and whole-cell sample and plotted using Uniform Manifold Approximation and Projection (UMAP). Both the whole-cell and longitudinal nanobiopsies showed separation between treated and untreated samples (Fig. 4B), and this separation was not due to confounding from any observed technical biases (figs. S22 and S23), suggesting that the nanobiopsies are capturing true biological effects of the treatment.

It has been shown previously that GBM cells lie on an axis of proneural (PN) to mesenchymal (MES) cell subtypes and that the distributions of these subtypes change through treatment (*29*). It is not, however, known whether the change in distributions is due to differences in growth and death rates of each subtype or instead due to cells directly changing subtypes through transcriptional reprogramming. To investigate this, we classified both treated and untreated M059K cells at each time point as either PN or MES (Fig. 4C) by applying gene set variation analysis (GSVA) to the nanobiopsy gene expression profiles. There was no difference in the propensity of cell subtypes at day 1, irrespective of treatment, to subsequently die (chi-square, *P* = 0.85) or survive (Fisher’s exact test, *P* = 0.67). There was also no difference, irrespective of treatment, in the proportion of cell subtypes at day 4 (Fisher’s exact test, *P* = 0.46). There was, however, a significant difference in the proportion of cells that switched subtype, or produced progeny with a different subtype when dividing, over time: of the cases where both day-1 and longitudinal biopsies had passed filtering, untreated cells switched subtype in 7 of 10 cases, whereas treated cells only switched in 1 of 9 (Fisher’s Exact Test, *P* = 0.0094; Fig. 4D). This suggests that untreated cells are significantly more plastic over the 3-day time course than treated cells, which is further indicated in the changes in both PN (absolute change in score is 3.0-fold higher in untreated versus treated cells; *t* test, *P* = 0.041) and MES scores (absolute change in score is 2.6-fold higher in untreated versus treated cells; *t* test, *P* = 0.028) over time (fig. S24). The distributions of phenotypes in both treated and untreated day-4 biopsies were similar to those from the whole-cell lysate (chi-square treated, *P* = 0.34, untreated *P* = 0.66; section S3.7).

## DISCUSSION

There is considerable interest in emerging technologies that can enhance our ability to understand and analyze transcriptome dynamics. Recent developments in in vivo fluorescence and super-resolution microscopy enabled the visualization of transcription dynamics in living cells (*5*, *41*, *42*). scRNA-seq and lineage tracing have been coupled to combine clonal information with cell transcriptomes (*43*, *44*), and metabolic labeling has been used to track newly synthesized RNA to allow the study of transcriptional dynamics to investigate how perturbations impact gene expression (*45*–*47*). However, understanding how the initial state of a cell’s transcriptome influences its response to a perturbation remains challenging due to the assumptions required to infer a cell’s initial state when using methodologies that require cell lysis (*10*). Recently, the development of Live-seq (*12*) enabled single-cell profiling and functional analysis of the same cell at distinct time points, preserving cellular functions and viability. The development of such technologies is of crucial importance to complement already available high-throughput techniques to unravel the highly dynamic mechanisms of gene expression dynamics and phenotype variation that characterize healthy and diseased tissues.

In this study, we developed a SICM-based technology using a double-barrel nanopipette to longitudinally profile the transcriptome of single cells in vitro over 72 hours. Such investigations allow for unprecedented insights into how cells respond under different conditions or with different treatments and will likely be invaluable in many research settings including, for example, in understanding the development of treatment resistance in cancer. This may be especially relevant in GBM, where, following surgical resection and subsequent treatment with radiotherapy and chemotherapy, tumors almost always recur and are fatal, resulting in a median patient survival of just 15 months (*48*). It is not known what allows the GBM cells to survive treatment, but recent studies suggest that therapy resistance is not driven by somatic mutations but is more likely owing to transcriptionally defined cell phenotypes that are inherently resistant or can reprogram to adapt and survive (*27*, *49*–*51*). These investigations have largely relied on comparing cell populations from distinct pre- and posttreatment tumor samples, and therefore, it has not been possible to gather direct evidence of transcriptional reprogramming, only to try and infer cell trajectories. We applied the nanobiopsy technique to M059K GBM cells to obtain sequential transcriptional profiles from the same cells as they undergo standard treatment, which includes radiotherapy and chemotherapy. The analysis of the sequencing metrics indicates that the nanobiopsy samples exhibit lower overall quality compared to the whole-cell lysate samples. This outcome was expected since the nanobiopsy samples represent only a small fraction of the total cytoplasmic mRNA, as supported by the estimated extracted volume (femtoliters) compared to the cytoplasmic volume (picoliters). Approximately, 53% of the nanobiopsy samples met the filtering criteria. Of these, a separation between treated and untreated longitudinal nanobiopsy samples was observed on a UMAP plot indicating that the mRNA extracted through nanobiopsy can be used to investigate changes in the cell’s transcriptional profile through treatment. The cell phenotype scores of paired day-1 and longitudinal samples revealed that treated cells tend to maintain the same phenotype during therapy, while untreated cells are more likely to switch transcriptional state over 72 hours, suggesting that treatment either induces or selects for higher transcriptional stability in this established GBM cell line. This use case, thus, offers an interesting insight into glioma biology which must be further investigated in additional GBM cell models.

While the nanobiopsy technology described in this study presents a powerful tool for longitudinally profiling the transcriptome of individual cells, it is essential to acknowledge the impact of the nanobiopsy process on cellular function. For example, the nanobiopsy could influence certain cell phenotypes and introduce confounding factors into the downstream analysis. For instance, it is plausible that specific cellular subtypes are more susceptible to damage or death following nanobiopsy, affecting the analysis of gene expression profiles. Limiting any impact on the cell phenotype caused by the nanobiopsy process will be paramount to facilitate data interpretation.

Future developments in the nanobiopsy technology will lead to an improved post-biopsy survival rate, along with an increase in the extracted volume coupled with an optimization of the protocol for reverse transcription and library preparation to enhance the quality of sequencing data. Furthermore, future studies will involve profiling the same cell at multiple time points, surpassing the longitudinal two–time point approach. These extended analyses will complement the state-of-the-art inference methods to measure cell trajectories, providing invaluable insights into the dynamic nature of gene expression. Moreover, this method allows for the spatially controlled injection of exogenous molecules into cells and could enable the sequential stimulation and monitoring of cellular responses for the spatiotemporal analysis of gene expression. We anticipate that the nanobiopsy technique will serve as a catalyst for the advancement of novel technologies in single-cell sampling, thereby expanding the rapidly evolving domain of temporal transcriptomics.

## MATERIALS AND METHODS

### Cell culture

HeLa (ECACC), CVCL-0030, and M059K (ECACC, ATCC CRL-2365) cells were cultured in Dulbecco’s modified Eagle’s medium/Nutrient Mixture F-12 (DMEM/F-12) (Gibco) supplemented with 10% fetal bovine serum (Gibco) and 1× penicillin/streptomycin solution (Life Technologies) in a 5% CO_2_-humidified atmosphere at 37°C and maintained at less than 80% confluence before passaging. The GFP-transfected M059K cell line was generated using the Dharmacon GIPZ Lentiviral shRNA library encoding the green-fluorescent protein TurboGFP, denoted as M059K_GFP_.

### Nanopipette fabrication

Double-barrel nanopipettes were pulled out of quartz theta capillaries (OD 1.2 mm, ID 0.9 mm, QT120-90-7.5, World Precision Instruments) using a CO_2_ laser puller (Sutter P2000, Sutter Instrument). A two-line program (Line 1: HEAT 750, FILAMENT 4, VELOCITY 30, DELAY 150, PULL 80/Line 2: HEAT 680, FILAMENT 3, VELOCITY 40, DELAY 135, PULL 160) was used to generate double-barrel nanopipettes with pore dimension of the individual barrel ~150 nm.

### SICM setup

The double-barrel nanopipette was mounted on a custom-designed holder. An Ag/AgCl and an Ag electrode were inserted in the aqueous and organic barrels, respectively. Each electrode was connected to a headstage amplifier (Axopatch CV-7B) mounted on the SICM frame. The headstage amplifiers were connected to a patch-clamp amplifier (MultiClamp 700B) and to an analog-to-digital converter (Digidata 1550B). The ion current was sampled using a sampling frequency of 10 kHz. The SICM setup consisted of an Axon MultiClamp 700B amplifier, an MM-6 micropositioner (Sutter Instrument), and a P-753 Linear actuator (Physik Instrumente) to allow precise three-dimensional movement of the nanopipette. The SICM software was used to control the positioning and topographical scanning capabilities of the SICM (ICAPPIC, London, UK). The *z*-piezo actuator had a travel range of 38 μm, while the travel range for *x* and *y* piezo was 96 μm. An Eclipse Ti2 inverted microscope (Nikon Instruments) and LED illumination system (pE-4000 CoolLED) with filter sets for DAPI, FITC, and TxRed were used for bright-field and epifluorescence imaging. An ORCA-Flash4.0 V3 Digital CMOS camera (C13440-20CU, Hamamatsu) was used to acquire optical and fluorescent micrographs.

### Longitudinal nanobiopsies

#### Oligos

Oligo-dT30VN: 5′-AAGCAGTGGT ATCAACGCAG AGTACTTTTT TTTTTTTTTT TTTTTTTTTT TTTTTVN-3′.

Template switch oligo (TSO): 5′-AAGCAGTGGTATCAACGCAG AGTACrGrG+G-3′ where “r” indicates a ribonucleic acid-base and “+” a locked nucleic acid base.

IS-PCR primers: 5′-AAGCAGTGGTATCAACGCAGAGT-3′.

All oligos were purchased from Integrated DNA Technologies.

#### Solutions

Lysis buffer was prepared in aliquots of 2 μl stored at −80°C and contained 0.04 μl of 10% Triton X-100 (Sigma-Aldrich), 0.1 μl of SUPERase·In RNAse Inhibitor (20 U/μl) (Thermo Fisher Scientific), and 1.86 μl of nuclease-free water for a total volume of 2 μl.

Mastermix 1 (priming mix) was prepared in aliquots of 19 μl (10 rxs) and stored at −80°C and contained 1 μl of dNTP mix (10 mM each, Thermo Fisher Scientific) and 0.9 μl of nuclease-free water (Thermo Fisher Scientific) for a total volume of 1.9 μl per reaction. Before use, 1 μl of Oligo-dT30VN (100 μM) was added to the thawed 19-μl aliquot for a total volume of 20 μl (10 rxs).

Mastermix 2 (RT-PCR mix) was prepared in aliquots of 48.5 μl (10 rxs) and store at −80°C and contained 0.06 μl of MgCl_2_ (1 M, Sigma-Aldrich), 2 μl of betaine (5 M, Sigma-Aldrich), 0.5 μl of DTT (100 mM, Thermo Fisher Scientific), 2 μl of 5× SuperScript first-strand buffer (Thermo Fisher Scientific), and 0.29 μl of nuclease-free water (Thermo Fisher Scientific) per reaction. Before use, 1 μl of TSO oligo (100 μM), 2.5 μl of SUPERase·In RNAse Inhibitor (20 U/ μl), and 5 μl of SuperScript II (200 U/ μl) were added to the thawed 48.5-μl aliquot for a total of 57 μl (10 rxs).

Mastermix 3 (IS-PCR) was freshly prepared and contained 12.5 μl of Kapa HiFi Hotstart Readymix (2×, Roche), 0.25 μl of IS PCR Primers (10 μM), and 2.25 μl of nuclease-free water for a total of 15 μl per reaction.

#### Cell preparation

M059K_GFP_ and M059K_WT_ cells were detached from the flasks using trypsin-EDTA solution (Sigma-Aldrich) and centrifuged at 1000 RCF for 5 min. Cells were resuspended in 1 ml of fresh medium and counted using a Hemocytometer (Burker, DHC-B01-50). M059K_GFP_ and M059K_WT_ cells were diluted to the same concentration and seeded into a 35-mm μ-Dish, high Grid-500 (IBIDI IB-81166) with seeding density 2.5 × 10^4^ cells per dish at a ratio of 1:350 (M059K_GFP_:M059K_WT_) to facilitate the identification and tracking of an individual M059K_GFP_ cell on the grid. After seeding, cells were incubated in a 5% CO_2_-humidified atmosphere at 37°C for 12 to 18 hours before nanobiopsy. Shortly before the experiment, the culture medium was replaced with 2 ml of phenol-free DMEM/F-12 (Gibco) supplemented with 10% FBS (Gibco) and 1× penicillin/streptomycin solution (Life Technologies).

#### Probe preparation

One barrel of the nanopipette was filled with 5 μl of 100 μM fluorophore (ATTO 488, 41051 Sigma-Aldrich, for HeLa and ATTO 565, 75784 Sigma-Aldrich, for M059K) in 0.1 M KCl (Sigma-Aldrich). The operation was performed ensuring that dry conditions were maintained to avoid water to wet the backend of the nanopipette which represents the main cause of cross-talk between the two barrels. The second barrel was filled with 5 μl of the organic phase mixture of 10 mM THATPBCl in 1,2-dichloroethane anhydrous (Sigma-Aldrich), following the silanization of the backend of the nanopipette by exposure to trichloro(1*H*, 1*H*, 2*H*, 2*H*-perfluorooctyl)silane (Sigma-Aldrich) for 10 s. This operation makes the glass hydrophobic and reduces the cross-talk during the experiment. After filling the two barrels, the nanopipette was left for 5 min at 60°C to ensure that the back of the nanopipette was dry before the experiment. The ion current generated in the aqueous barrel was used as SICM feedback to drive the nanopipette toward and inside the cell. The electrolyte solution in the aqueous barrel has high electrical conductivity (σ_aq_ = 1.3 S/m) and generates an ion current with greater magnitude than the one generated in the organic barrel, where the conductivity of the organic solution is two orders of magnitude smaller (σ_org_ = 0.011 S/m) (*30*).

#### Nanobiopsy

An individual M059K_GFP_ was identified on the grid using a 10× magnification objective (Nikon) in epifluorescence microscopy, ensuring that no other M059K_GFP_ cells were detected in the same field of view. Next, a 40× objective (Nikon) was used to acquire the fluorescence and optical micrograph of the cell before nanobiopsy. The double-barrel nanopipette was mounted on the SICM holder and was immersed using the linear actuator of the SICM until an ion current through the aqueous barrel was detected. During immersion, the potential applied to the electrode in the aqueous and organic barrel was *V*_aq_ = 200 mV and *V*_org_ = 300 mV, respectively, with respect to a reference Ag/AgCl electrode immersed in the culture media. In the approach phase, the SICM working in hopping mode (HPICM) (*34*) moved the double-barrel nanopipette toward the cell membrane until the set point current (99.5% of reference current) was detected in the aqueous barrel. At this point, the system stopped the nanopipette and retracted it to a distance from the cell equal to the hopping height parameter that was set to 2 μm. Following the approach, the double-barrel nanopipette was manually positioned onto the perinuclear region of the cell by means of a micromanipulator. In the nanoinjection phase, the double-barrel nanopipette was first moved vertically toward the cell of a distance equal to the hopping height (2 μm) to reach a position that is estimated to be <500 nm from the cell membrane. Next, the potential applied to the electrode in the aqueous barrel was switched to *V*_aq_ = −500 mV to release the anionic fluorophore and the double-barrel nanopipette was moved toward the cell vertically with 100-nm steps at 10 nm/ms until the detection of the vibrational noise due to the proximity of the double-barrel nanopipette to the polymer coverslip of the dish. Upon detection of the vibrational noise, the indentation of the nanopipette to the cell membrane is enough to puncture the membrane and access the cytoplasm. Next, the nanopipette was withdrawn at 100 nm and was kept in position for ~60 s to allow the electrophoretic release of the anionic fluorophore into the cytoplasm. At the end of the nanoinjection phase, the cell emitted a red fluorescent signal that was visualized in epifluorescence. Following nanoinjection, the cytoplasmic extraction is enabled by switching the potential applied to the electrode in the organic barrel to *V*_org_ = −500 mV for 10 s. The application of the negative potential generated an inflow of cytoplasm in the nanopipette via electrowetting of the organic phase in the nanopipette barrel. A successful cytoplasmic extraction resulted in an increased magnitude of the ion current in the organic barrel *i*_org_ due to the ingress of cytoplasm whose conductivity (σ_cytoplasm_ ~ 0.35 to 0.5 S/m) (*52*) is 30 to 45 times greater than the conductivity of the organic solution (σ_org_ = 0.011 S/m). Following cytoplasmic extraction, the double-barrel nanopipette was withdrawn and unloaded from the SICM and immediately placed in a tube preloaded with 2 μl of lysis buffer containing an RNAse inhibitor to prevent mRNA degradation. The tube was immersed in liquid nitrogen and stored until reverse transcription and cDNA amplification via Smart-Seq2 which was performed in all cases <7 days following nanobiopsy. The time elapsed from the withdrawal of the nanopipette to the immersion of the tube in liquid nitrogen was estimated to be <3 min, including the time required to withdraw the nanopipette from the culture solution (~30 s), remove the electrode and unload the nanopipette (~ 30 s), gently break the tip in the tube containing lysis buffer (~30 s), and immerse the tube in liquid nitrogen (<30 s). Following nanobiopsy, the dish containing the biopsied cells was left to recover in a 5% CO_2_-humidified atmosphere at 37°C for 24 hours. On day 2, the dish was either subjected to nonsurgical elements of standard GBM treatment consisting of 2-Gy IR and 30 μM TMZ (treated samples) or to mock IR (untreated samples). On day 3, cells were allowed to recover in the incubator. On day 4, the same cell or the progeny thereof was localized in the same location on the grid in epifluorescence and bright-field microscopy, and the same nanobiopsy procedure was carried out to perform longitudinal cytoplasmic extractions. Nanobiopsies were conducted over various passages of the M059K cell line, distributed across separate days, to mitigate potential technical bias originating from batch effects. Specifically, day-1 and longitudinal nanobiopsies were carried out over 20 and 16 distinct days, respectively. The number of nanobiopsies performed ranged from a minimum of 1 to a maximum of 16 nanobiopsies per day.

#### Reverse transcription and cDNA amplification

The Smart-seq2 protocol (*38*) was adjusted to optimize the reverse transcription and cDNA amplification of the mRNA extracted via nanobiopsy. An aliquot of Mastermix 1 and Mastermix 2 were thawed on ice and the required reagents were added to complete the reaction mix. A volume of 2 μl of Mastermix 1 was dispensed in the PCR tube containing the nanobiopsy sample in 2 μl of lysis buffer. Next, the tube was vortexed and briefly centrifuged. The tube was incubated in a thermocycler (Eppendorf Mastercycler Nexus GX2) at 72°C for 3 min to anneal the Oligo-dT30VN to the poly-A tail of the mRNA transcripts. After incubation, the tube was immediately placed on ice for 1 min. Next, the tube was centrifuged for a few seconds to collect condensations. A volume of 5.5 μl of Mastermix 2 was dispensed into the PCR tube. Next, the tube was vortexed and briefly centrifuged. The total reaction volume was ~10 μl. The following thermal cycling program was performed using a thermocycler: (1) 42°C for 90 min; (2) 50°C for 2 min; (3) 42°C for 2 min, go to step (2) for 10 cycles; (4) 70°C for 15 min and (5) 4°C hold. The cDNA resulting from the reaction was amplified using a hot-start PCR to obtain the concentration required for library preparation. Fifteen microliters of Mastermix 3 was added to the tube containing the RT-PCR product for a total reaction volume of ~25 μl. The following thermal cycling program was performed using a thermocycler: (1) 98°C for 3 min; (2) 98°C for 20 s; (3) 67°C for 15 s; (4) 72°C for 6 min, go to step (2) for 26 cycles; (5) 72°C for 5 min; and (6) 4°C hold. The amplified cDNA was purified using the HighPrep PCR Clean-up System (Sigma-Aldrich, AC-60050) adding a volume of magnetic beads equal to 0.8× the sample volume. The individual cDNA samples were quantified using the fluorometric assay QuantiFluor dsDNA System (Promega, E2670) using a 500–base pair dsDNA calibration sample. The quality and fragment size of the amplified cDNA were quality-checked using a D5000 tape (Agilent).

#### Library preparation and next-generation sequencing

Next-generation sequencing libraries were prepared using the Nextera XT DNA Library Preparation Kit (Illumina, FC-131-1096). Following fluorometric quantification, each cDNA sample was diluted to a concentration of 0.2 ng/μl, which is the concentration needed for the tagmentation reaction. cDNA samples were diluted in a 96-well plate (plate 1: cDNA). The concentration of 12 wells (diagonal) of the plate containing the diluted cDNA was checked using the QuantiFluor dsDNA System (Promega, E2670) fluorometric assay as the initial cDNA concentration is a critical step to obtain libraries with adequate fragment length. The tagmentation mastermix consisting of 300 μl of Tagment DNA Buffer and 150 μl of Amplicon Tagment Mix for a total volume of 450 μl was prepared on ice and a volume of 3.75 μl of the tagmentation mastermix was dispensed into each well of a new 96-well plate (plate 2: tagmentation) on ice. Next, 1.25 μl of the diluted cDNA (0.2 ng/μl) was transferred from plate 1 to plate 2. Plate 2 was sealed and centrifuged at 1000*g* for 1 min at 4°C and the tagmentation reaction was performed using a thermocycler as follows: (1) 55°C for 10 min and (2) 10°C hold. Next, 1.25 μl of Buffer NT and 3.75 μl of Nextera PCR Mastermix (NPM) from the Illumina kit were added to each well of the 96-well plate on ice. For each well, a total of 2.5 μl of Illumina indexes (i5 index + i7 index) were added at a 1:1 ratio. Following indexing, the amplification of the tagmented DNA fragments was performed using a thermocycler: (1) 72°C for 3 min; (2) 95°C for 30 s; (3) 95°C for 15 s; (4) 55°C for 10 s, go to step (3) for 12 cycles; (5) 72°C for 5 min; and (6) 4°C hold. At the end of the PCR reaction, the cDNA fragments are of ideal size and uniquely barcoded (library). The concentration of the individual libraries was spot-checked on the diagonal of the 96-well plate (12 samples) to ensure that the library concentrations were not too different from each other. A multiplexed library was generated by transferring 5 μl of each individual library into a tube and the multiplexed library was purified using the HighPrep PCR Clean-up System using a library:bead ratio of 1:0.6. The size of fragments of the multiplexed library was checked on a D1000 tape (Agilent). All the multiplexed libraries generated were sequenced using NextSeq2000 (Illumina), a high-throughput flow cell, and adopting a P2 200 cycle (100 Pair End) and P3 200 cycle (100 pair-end) strategy.

### Whole-cell lysate preparation

The M059K_GFP_ cells were grown to 80% confluency inside the tissue culture flask, the cells were then separated into two flasks by depositing the same number of cells and allowed to grow 50% confluency in both flasks before the next step. One of the two flasks was then subjected to a treatment of 2-Gy IR and 30 μM TMZ. These cells were labeled as “treated cells,” while the control flask of cells was labeled as “untreated cells.” Both flasks were left inside the incubator for 24 hours before fluorescence-activated cell sorting (FACS). FACS was used to sort the viable M059K_GFP_ into a 96-well plate. Two microliters of lysis buffer consisting of 0.2% Triton X-100 and SUPERase·In RNAse Inhibitor (1 U/μl; Thermo Fisher Scientific) was added to all the wells of the 96-well plate. This procedure was carried out inside a RNAse decontaminated laminar flow hood. Before FACS, the treated cells were harvested and washed twice with ice-cold phosphate-buffered saline at the concentration of 0.5 × 10^6^ cells/ml and stained with DAPI (Sigma-Aldrich) at the concentration of 0.1 μg/ml to exclude dead cells. For FACS, the gates were adjusted to exclude cell debris and doublets by size and scatter selection, only the viable GFP-positive cells were selected, and subsequently single-cell–sorted into the lysis buffer containing a 96-well plate. The sorted plates were immediately sealed, centrifuged at low speed for 1 min, snap-froze with dry ice, and stored at −80°C. The same harvesting and FACS procedures were used again to single-cell–sort the untreated cells on the same day. The plates were used within 2 weeks after the sorting. The single-cell–sorted plates were subjected to the adjusted Smart-Seq2 protocol (*38*) as outlined in the longitudinal nanobiopsy procedure with the exception that all the reaction solutions were freshly prepared before the procedure. The amplified cDNA samples were then used for library preparation as outlined in the longitudinal nanobiopsy procedure above.

### Data analysis

Reads were trimmed with Cutadapt (v4.1) to remove Nextera adapters and low-quality bases with the parameters “-a CTGTCTCTTATA -A CTGTCTCTTATA --minimum-length 30 --overlap 5 -q 10”. Reads were then aligned and transcripts quantified using the GRCh38 genome with Gencode v27 basic annotations via STARsolo (v2.7.10b) with “-outFilterMatchNmin” set to 60 to ensure high stringency alignments. The resulting BAM files were split using SAMtools split (v1.16.1) and sequencing metrics were generated via Picard CollectRnaSeqMetrics (v2.20.2-SNAPSHOT). Nanobiopsies and whole-cell lysates datasets were filtered for >150 expressed genes, <30% mitochondrial bases, and < 10% ribosomal bases. Genes were filtered separately in each dataset for those that have >3 counts in each of two or more cells. Non–protein-coding genes were also removed. Gene counts were normalized and scaled via Seurat (v4.3.0) using default settings with the addition of “scale.max = 5” and regressing out number of genes expressed and % mRNA bases, for the nanobiopsies, and number of genes expressed for the whole-cell lysates. The top 600 highly variable genes were identified in each using the Seurat “disp” method and used to perform PCA. UMAPs were then generated from the first eight principal components. GSVA (v1.42.0) was run on the nanobiopsy-scaled gene expression. Gene sets for MES and PN phenotypes were taken from the top and bottom 50 weighted genes from PC1 for the 10× single-cell dataset in (*29*). The stemness gene set was taken from (*53*). All statistical tests performed were two-tailed.

## Supplementary Materials

### This PDF file includes:

Sections S1 to S3 Figs. S1 to S24
