# Supplementary material for: Single-cell nanobiopsy enables multigenerational longitudinal transcriptomics of cancer cells
Source: Sci Adv. 2024 Mar 6;10(10):eadl0515. doi: 10.1126/sciadv.adl0515 (PMC10917339; doi:10.1126/sciadv.adl0515)
Supplement: Supplementary file 1 — Sections S1 to S3 Figs. S1 to S24 [file sciadv.adl0515_sm.pdf]

Science Advances
AAAS

Supplementary Materials for

Single-cell nanobiopsy enables multigenerational longitudinal transcriptomics of cancer cells

Fabio Marcuccio *et al.*

Corresponding author: Lucy F. Stead, l.f.stead@leeds.ac.uk; Paolo Actis, p.actis@leeds.ac.uk

Sci. Adv. **10**, eadl0515 (2024)
DOI: 10.1126/sciadv.adl0515

This PDF file includes:

Sections S1 to S3
Figs. S1 to S24

1: Single-cell nanobiopsy technique

1.1 Nanopipette fabrication and characterization.

Double-barrel nanopipettes used in this work were fabricated from theta-quartz capillaries using a Sutter P-2000 laser puller using the following parameters:

	HEAT	FILAMENT	VELOCITY	DELAY	PULL
**LINE 1**	750	4	30	150	80
**LINE 2**	680	3	40	135	160

The SEM images and the measured dimension of the individual barrels for four different nanopipettes in **Fig. S1** show that one barrel (barrel 1) is characterized by a bigger aperture than the aperture of the other barrel (barrel 2) which is due to the asymmetric pulling process of quartz-theta capillaries.

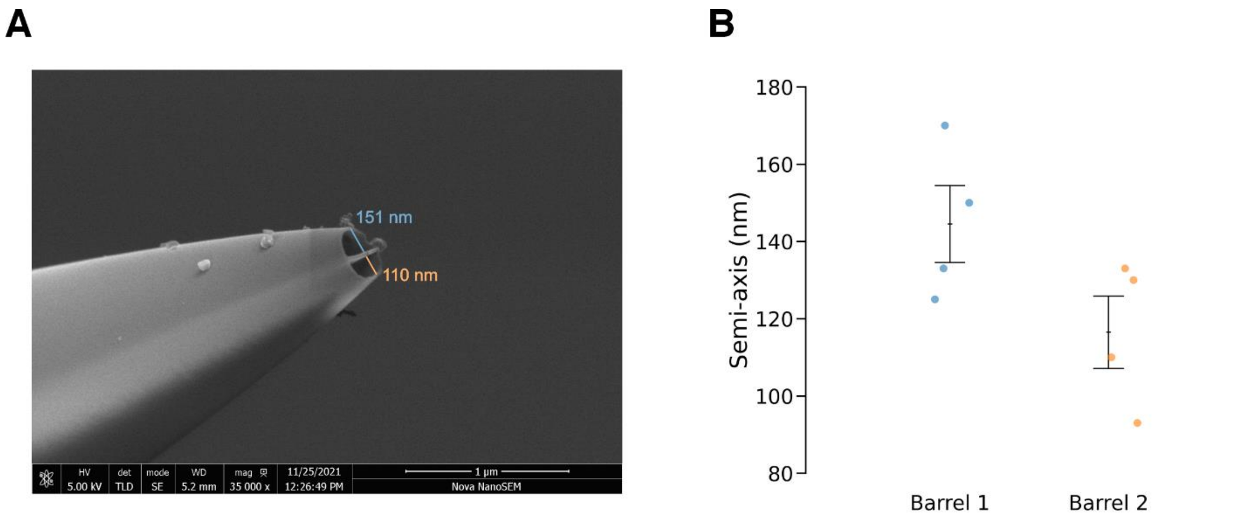

Figure S1: Double-barrel nanopipette characterization. (A) Scanning electron microscopy (SEM) micrograph showing the double-barrel nanopipette used in this work and (B) scatter plot showing the dimension of the semi-axis of each barrel across four different nanopipettes where barrel 1 is the barrel with larger aperture and barrel 2 is the barrel with smaller aperture. Error bars indicate the standard error of the mean.

1.2 Electrical resistance of the aqueous and organic barrel

The electrical resistance of the barrel filled with the aqueous solution of 0.1 M KCl and 100 µM ATTO 565 and the one filled with the organic solution of 10 mM THATPBCl in 1,2 DCE was calculated from the current trace of the aqueous and organic barrel using Ohm's law. For the aqueous barrel (**Fig. S2A**), the median value for the electrical resistance was 45.7 MΩ. For the organic barrel (**Fig. S2B**), the median value for the resistance was 2.7 GΩ which is ~59 times higher than the resistance of the aqueous barrel. In both cases, the total number of data points is 256.

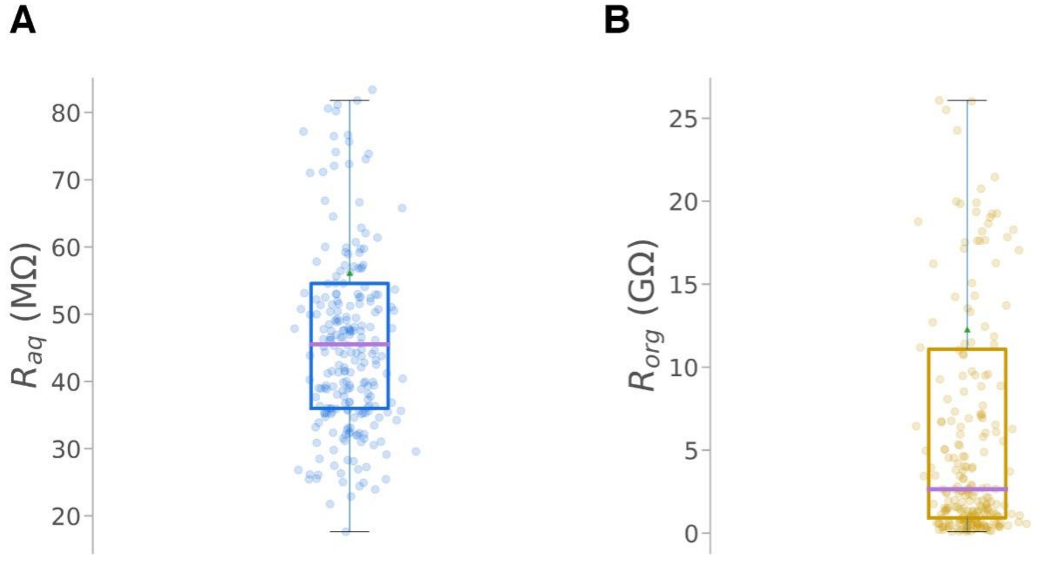

Figure S2: Electrical characterization of the individual barrels. Experimental electrical resistance of the aqueous (A) and organic (B) barrel of the nanopipettes used in the nanobiopsy experiments. Note the unit for R_{org} is GΩ. The green triangle and the purple line show the mean and median value, respectively. N = 256.

1.3 Example of electric signals recorded during the approach phase

In the approach phase (**Fig. S3A**), the double-barrel nanopipette automatically approaches the cell membrane driven by an SICM working in hopping mode with value of the setpoint current set to 99.5% of the baseline current and hopping height set to 2 µm. The approach of the cellular membrane is concluded when the current signature of the hopping mode shown in **Fig. S3B** is detected in the aqueous barrel, where the current i_{aq} drops to the setpoint current when the piezo actuator z reaches the hopping height value. At the end of the approach phase, the nanopipette is visible as a bright dot and it is manually moved to the biopsy location on the cellular membrane using an X-Y stage micromanipulator (**Fig. S3C-D**).

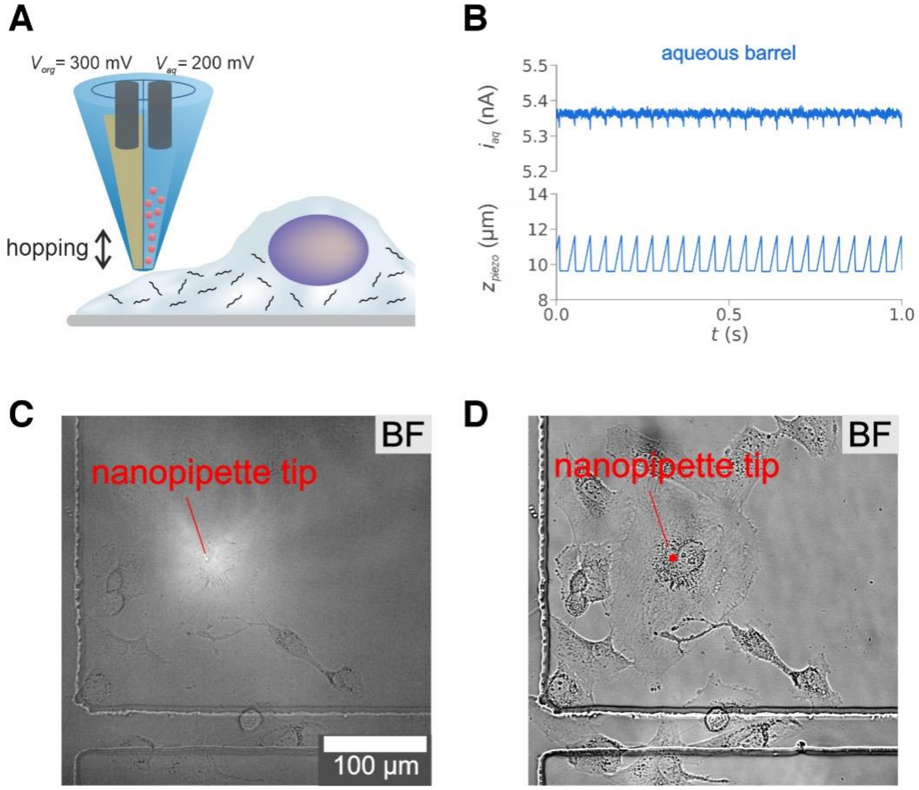

Figure S3: Illustration of a double-barrel nanopipette approaching the cellular membrane. (A) and experimental recordings of the ion current in the aqueous barrel i_{aq} and piezo vertical position z_{piezo} at the end of the approach phase (B), when the nanopipette tip appears as a bright dot under bright field (BF) microscopy (C). The position of the nanopipette after nanopipette withdrawal is marked with a red dot (D).

1.4 Power spectral density of the ion current in the nanoinjection phase

The trace of the ion current recorded in the aqueous barrel shown in **Fig. 2 A-B (main text)** can be analysed in the frequency domain to provide information on all the three sub-phases of the cell membrane and nanoinjection phase of the nanobiopsy experiment. **Figure S4** shows the spectrogram of the ion current trace i_{aq} shown in **Fig. 2A-B (main text)** with the z-piezo trace plotted at the top (the z-piezo signal was flipped for ease of representation). The hopping phase (1) can be identified by the high power of the frequencies multiple of 25 Hz (hopping frequency) in the first second of the map. Next, at t~5 s, a sudden increase in the power of all frequencies is observable upon the motion of the nanopipette from the hopping height (2 μm) to the surface of the cell (membrane touch phase (2)). The noise increase in the lower frequencies (<1000) is due to the proximity of the nanopipette to the plasma membrane. Next, the potential to the electrode in the aqueous barrel is switched to -500 mV and a spike in all frequencies can be observed around t=10 s. After the potential switch, the nanopipette is lowered down with 100-nm steps until the vibrational noise (3) is observable as an increase in the power across all frequencies around t=38 s. At the point where the piezo reaches the maximum extension (t=46 s), the power of all frequencies decreases of several orders of magnitude for approximately 3 seconds. The decrease in the power is due to the ion current having magnitude equal to 0 in this time interval, due to the contact between the nanopipette and the surface of the petri dish. This is the point of maximum indentation. Upon the nanopipette retraction of 100 nm, the ion current is re-established, and the spectrogram shows an increase in the power of all frequencies. Interestingly, after nanopipette retraction, the current continues to be characterized by higher power across all the spectrum (higher noise) that might be due to the cell cytoplasm which represents a noisier environment than the cell medium. The spectrogram of the ion current in the aqueous barrel of the nanopipette provides a summary of the cell membrane and nanoinjection phase. Compared to the time-domain signal, the spectrogram summarizes all the information in a map that can be used in a post-processing to extract and compare the parameters describing the experiment. A post-processing analysis of

these maps could help to identify the current signatures that could distinguish a successful from an unsuccessful nanobiopsy. For example, the spectrogram in **Fig. S5A** shows the three independent phases for the membrane penetration and nanoinjection resulting in the injected cell emitting a red-fluorescent signal as shown in **Fig. S5B.** As opposite, the spectrogram in **Fig. S5C** shows an irregular pattern with some intervals with no power in all frequencies because of the current being out of range of the analog-to-digital converter, resulting in the cell emitting no red fluorescent signal at the end of the procedure, as shown in **Fig. S5D,** suggesting that the nanopipette failed to penetrate the cellular membrane.

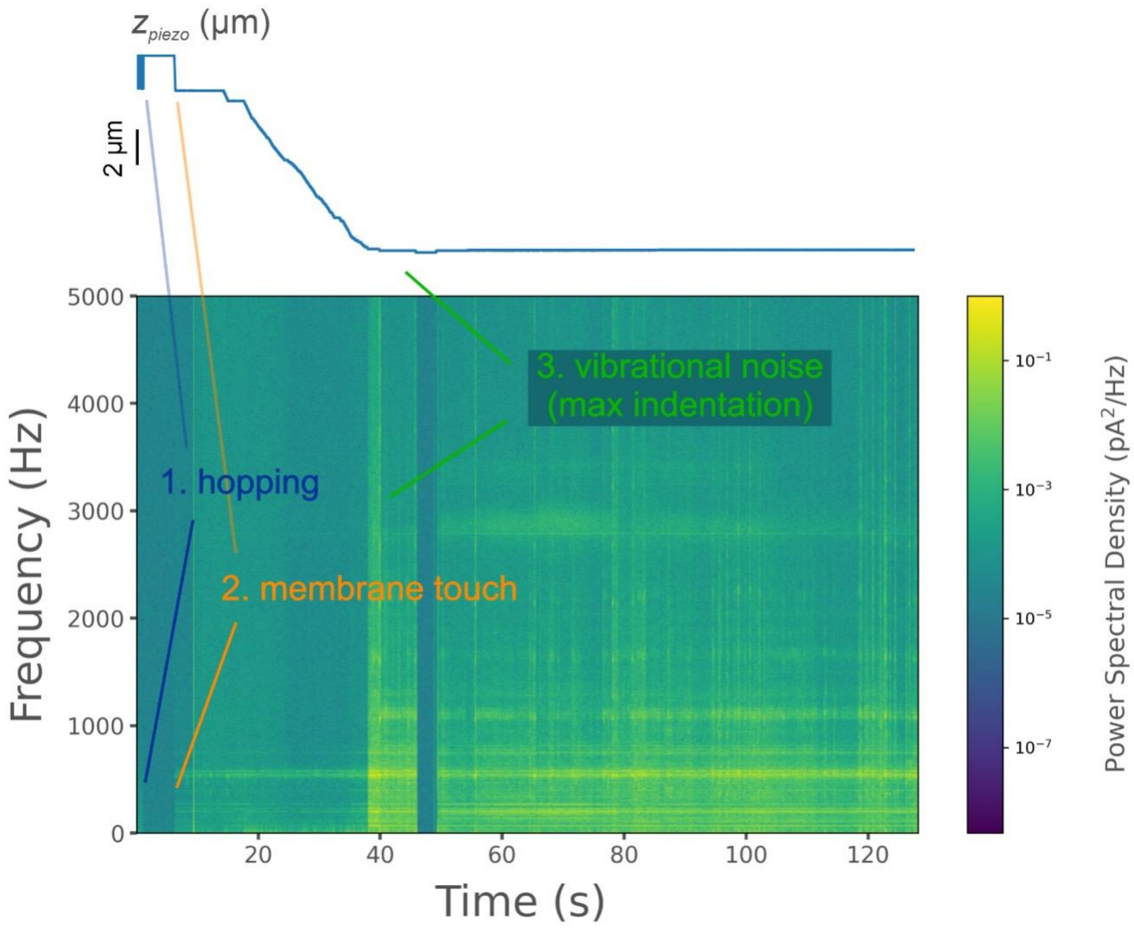

Figure S4: Spectrogram for the ion current recorded during the membrane penetration and nanoinjection phase in a M059K cell. The z-piezo trace is shown at the top for reference, and it was flipped to get a decrease in magnitude when the nanopipette is lowered towards the cell cytoplasm. The power spectral density (pA^2/Hz) is shown by the color map for each frequency over time. A frequency signature identifies the three main phases of the cell penetration and injection experiment including hopping (1), membrane touch (2) and vibrational noise (3). The spectrogram was generated calculating the Fast Fourier Transform using Hamming window with length equal to 2048 samples (corresponding to 0.2 s) and an overlap of 256 samples. For a signal sampled with 10 kHz sampling frequency, the parameters used generate a spectrogram with time resolution 0.2 s and frequency resolution 5 Hz.

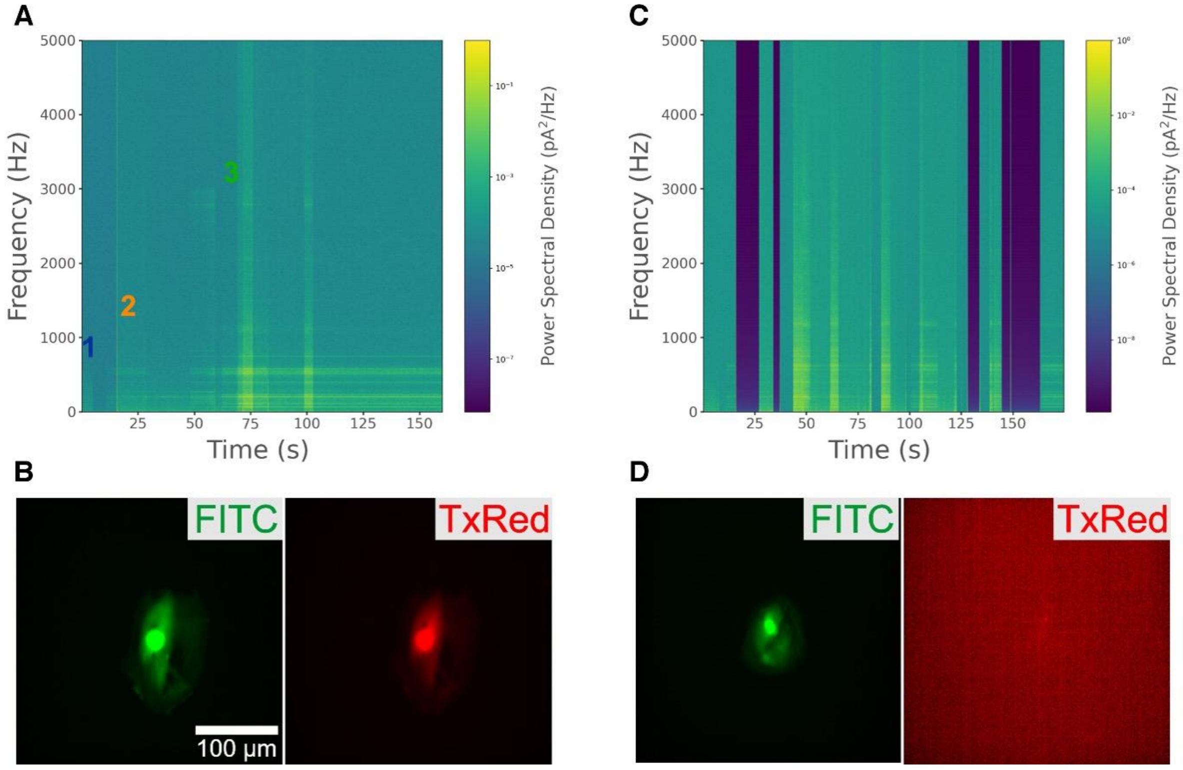

Figure S5: Spectrograms distinguish successful from unsuccessful cell membrane penetration and nanoinjections. Example of a spectrogram of the ion current signal in case of a successful (A-B) and unsuccessful (C-D) cell membrane penetration and nanoinjection. A spectrogram where all three sub-phases (i.e. hopping 1, membrane touch 2, vibrational noise 3) can be identified (A) results in the cell emitting a red-fluorescent signal following nanoinjection of a red fluorophore (B). A spectrogram with irregular pattern where the individual sub-phases cannot be identified (C) results in the cell emitting no red fluorescent signal at the end of the procedure (D).

1.5 Reproducibility of experimental traces during the nanoinjection phase

The spectrogram for the ion current in the aqueous barrel i_{aq} during the nanoinjection phase can be used as quality control to check whether the nanopipette was successful at penetrating the cellular membrane and accessing the cytoplasm. **Figure S6** shows some representative spectrograms recorded during the nanoinjection phase in the nanobiopsy of M059K$_{GFP}$ GBM cells where the three subphases, namely hopping (1), membrane touch (2) and vibrational noise (3), were detected and the red fluorescent molecule was successfully injected as observable in the optical micrographs.

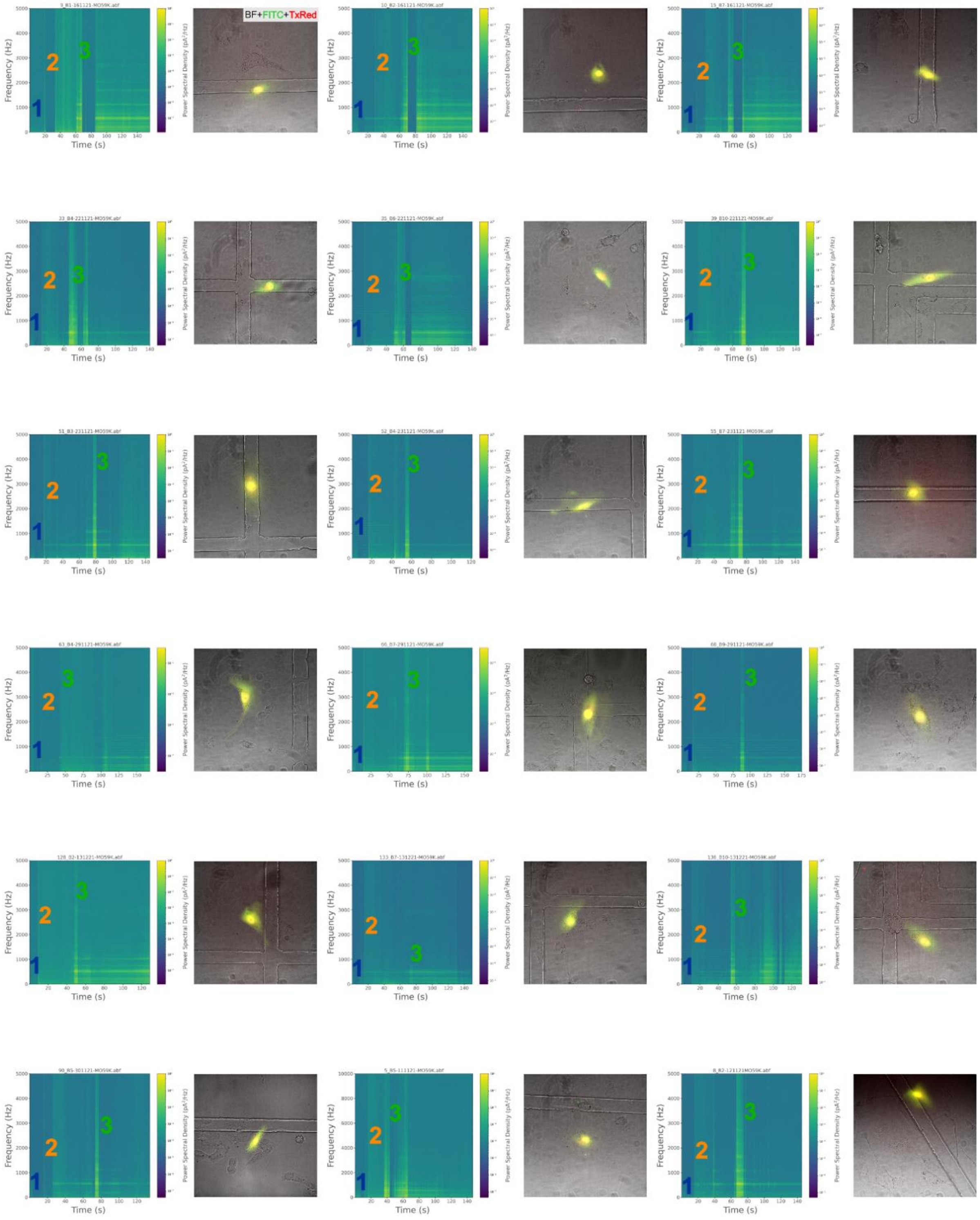

Figure S6: Representative spectrograms of the ion current in the aqueous barrel i_{aq} recorded during the nanoinjection phase and optical micrograph of the cell after nanoinjection. The three individual sub-phases hopping (1), membrane touch (2) and vibrational noise (3) can be identified in all spectrograms and can be used as an indication that the cellular cytoplasm was successfully accessed, as confirmed by the red fluorescent signal emitted by the cell after the procedure (yellow false color obtained by merging bright field (BF), green (GFP) and red (ATTO565) channels). Spectrograms were calculated from the ion current in the aqueous barrel i_{aq} recorded during 18 M059K$_{GFP}$ nanobiopsies performed on 8 different days. Each nanobiopsy was performed using a different nanopipette.

1.6 Success rate for membrane penetration and nanoinjection

A successful nanoinjection is defined as a nanoinjection that results in the cell emitting a clearly distinguishable fluorescent signal. The success rate for membrane penetration and nanoinjection was calculated across 10 experimental days. The average success rate across these days was 0.89 ± 0.07. On a typical experimental day, the success rate ranged from a minimum of 0.79 to a maximum of 1. The barplot in **Fig. S7** illustrates the success rate calculated for each of these 10 experimental days.

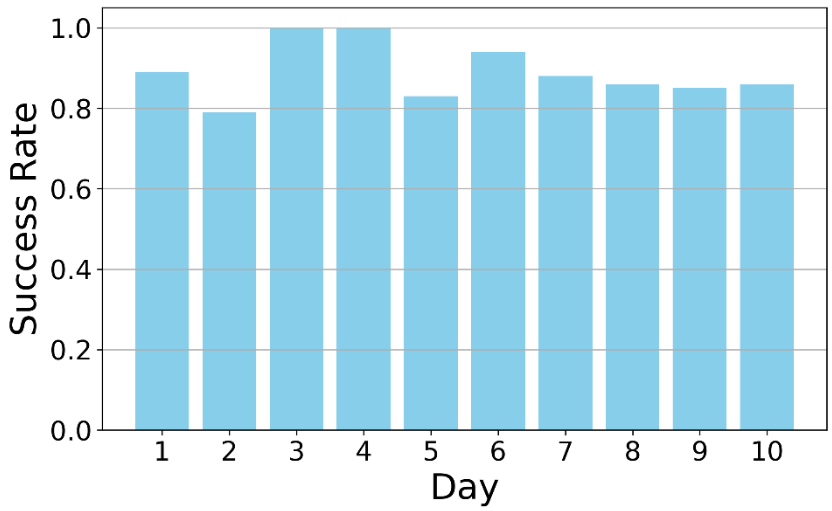

Figure S7: Success rate for membrane penetration and nanoinjection calculated across 10 experimental days.

1.7 Reproducibility of experimental traces during the nanobiopsy phase

The trace for the ion current in the organic barrel i_{org} can be used to assess whether a cytoplasmic volume was extracted in the nanobiopsy phase. If the value for i_{org} after the potential switch V_{org}=-500 mV for 10 s is greater than the value before the potential switch, a cytoplasmic volume is extracted. The ingress of cytoplasm in the nanopipette decreases the nanopipette resistance thus increasing the ion current i_{org} in the organic phase. **Figure S8** shows the ion current i_{org} recorded from 24 randomly selected nanobiopsies.

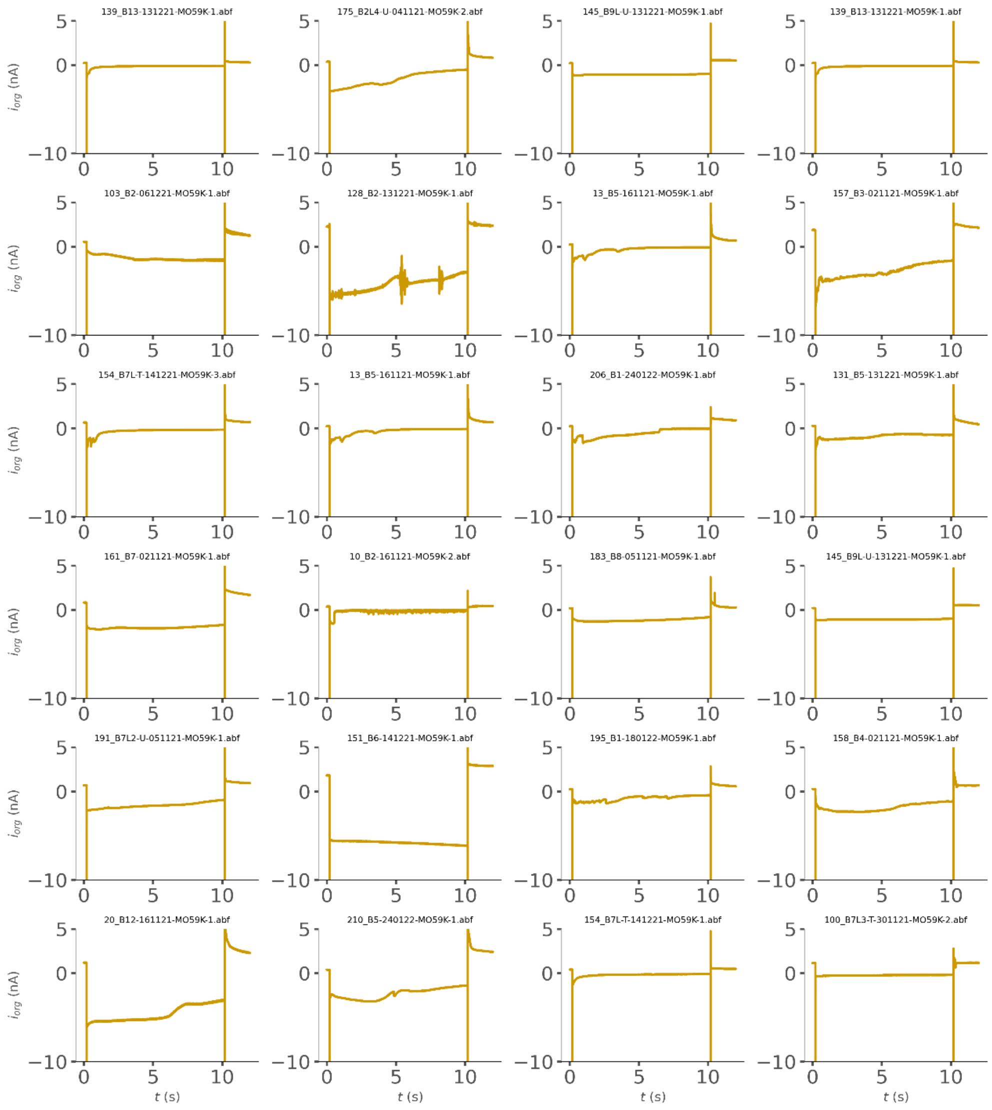

Figure S8: Representative traces for the ion current i_{org} in the organic barrel during the nanobiopsy phase. The potential applied to the electrode in the organic barrel during nanobiopsy is V_{org}=-500 mV for a duration of 10 s.

2: Longitudinal nanobiopsies of individual glioblastoma cells

2.1 Determination of cell topography

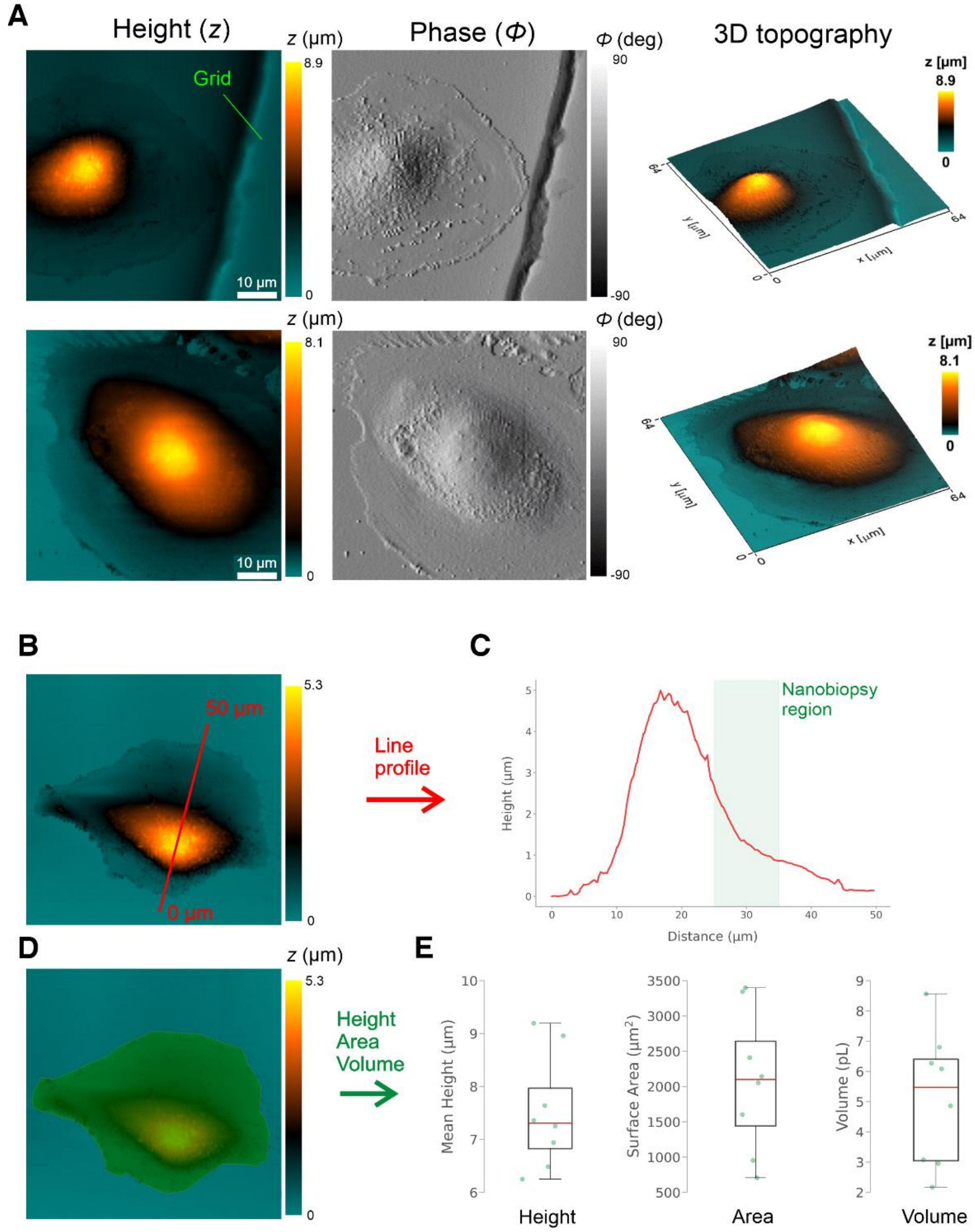

Figure S9: Determination of cell topography and morphological parameters using SICM. (A) Surface topography measurements of live glioblastoma cells using SICM. Topographical maps were reconstructed using the height (left) and phase (center) channel. The height channel was used to generate a 3D image (right). A channel of the grid on the dish is visible in the topographical map of the first cell (top). (B) Topographical map of a single glioblastoma cell where a 50 μm line segment was traced (red) over the cytoplasm and nucleus to measure the cell height profile (C). The high-slope region (green shaded area) was selected as suitable for nanobiopsy due to the slope facilitating membrane penetration. (D) Cellular area used to extract the mean height, surface area and volume whose values are shown in the boxplots (E) for a total number of cells equal to 8.

2.2 Fluorescent staining of fixed M059K GBM cells

Different combinations of dyes were used to image the organelles in the perinuclear region which was determined the most suitable region for the nanobiopsy. M059K cells were fixed using 4% paraformaldehyde and combinations of DAPI, Concanavalin A (Con A), MitoTracker green and cellMask orange were used to stain and image nuclear DNA, endoplasmic reticulum (ER), mitochondria and plasma membrane, respectively. **Figure S10** shows the fluorescent micrographs obtained by combining DAPI, Con A, MitoTracker (A), DAPI, Con A (B) and DAPI, cellMask Orange (C).

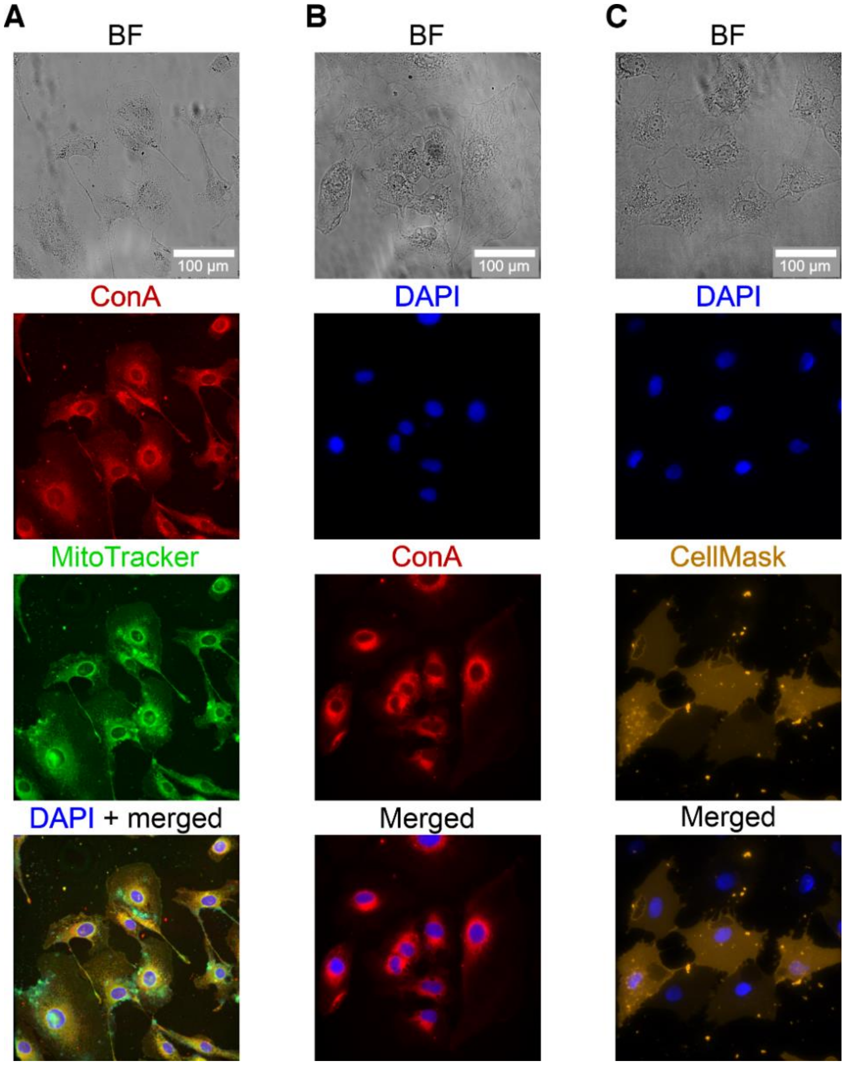

Figure S10: Fluorescent staining of fixed M059K GBM cells. From top to bottom, (A) bright field BF, Con A, MitoTracker, merged ConA and MitoTracker and DAPI for nuclear DNA, endoplasmic reticulum and mitochondria visualization. (B) BF, DAPI, ConA for nuclear DNA and endoplasmic reticulum visualization. (C) BF, DAPI, cellMask orange for nuclear DNA and plasma membrane visualization. Images were acquired using the BF, DAPI, TxRed and FITC filter sets.

2.3 Experimental setup and longitudinal tracking of individual M059K GBM cells

The experimental approach introduced in **Fig. 4A** (**main text**), requires tracking the same cell over the time course of 72 hours. To assess whether a mixed culture of $M059K_{GFP}$ and $M059K_{WT}$ and the use of gridded dishes could be used for this purpose, we measured the cellular migration over 4 days. $M059K_{GFP}$ and $M059K_{WT}$ GBM cells were plated with a 1:350 ratio ($M059K_{GFP}$: $M059K_{WT}$) according to the experimental methods in three different gridded dishes with the same seeding density ($2.5 \cdot 10^4$ cells/dish) on day 0 of the experiment. 24 hours later (day 1), an individual $M059K_{GFP}$ was identified and imaged in a specific location of the grid, ensuring that only one $M059K_{GFP}$ was present in the field of view imaged with a 10x magnification objective. After imaging, the dish was brought back to the incubator and the same location was imaged again after 24 (day 2), 48 (day 3) and 72 hours (day 4). The gridded dish enabled tracking of the same cell over time by finding back the same labelled position on the grid where the $M059K_{GFP}$ cell was identified on day 1. **Figure S11A** shows one example of an individual $M059K_{GFP}$ cell imaged on day 1 in position A11 on the grid. After 24 hours (day 2), the same cell was found back by imaging the same dish location (A11). Two cells were found on day 2, suggesting that the cell went through cell division. After 48 (day 3) and 72 hours (day 4) the same cells were found back in the same location. On day 4, four cells were found around A11, suggesting that both cells found on day 2 and day 3 divided. An image processing routine was developed to measure different cell migration parameters:

- The **total cell migration** Δx_{tot} is the maximum distance travelled by the cell over 72 hours and it was calculated as the sum of the longest distance (even if the least likely) travelled by the cell every 24 hours (Δx_1, Δx_2, Δx_3**)**, as shown in **Fig. S11B**
- The **maximum cell migration** Δx_{max} was calculated as the length of the segment (orange) connecting the point indicating the cell on day 1 (purple) to the farthest point indicating the farthest cell on day 4 (red), as shown in **Fig. S11C**

- The **total area enclosing all cells after 72 hours A_{all}** was measured by tracing the smallest rectangle enclosing all the points/cells of all days (purple, green, blue, red), as shown in **Fig. S11D**
- The **mean maximum migration over 24 hours** was measured by considering the mean of the longest distance travelled by the cell every 24 hours over 72 hours **($\Delta x_1 + \Delta x_2 + \Delta x_3$)/3**
- The **field of view (FOV)** is defined as the observable area under the microscope through the camera using a 10x objective. The field of view shown in **Fig. S11E** (black) has size 1300 x 1300 µm and area 1.69 mm^2.

The parameters listed above were measured for three cells across three different dishes. **Figure S11F-I** shows the results obtained for each of these parameters. For all parameters, no statistical significance was found between different replicates (t-test, $p<0.05$) except from the total migration between dish 1 and dish 2 which showed a slight significance ($p=0.05$). The total migration Δx_{tot} (**Fig. S11F**) and the maximum migration Δx_{max} (**Fig. S11G**) show that the longest distance that a cell can travel in the worst-case scenario is always significantly smaller than the dimensions of the field of view. This suggests that a cell is not able to migrate out of the field of view in the time course of the experiment. The enclosing area A_{all} (**Fig. S11H**) was found in all cases to be significantly smaller than the area of the field of view. The biggest A_{all} found had value 0.35 mm^2 which is ~5 time smaller than the area of the field of view (1.69 mm^2), suggesting that all cells (initial cell and progeny) remain within the field of view area in the time course of the experiment. The mean migration over 24 hours (**Fig. S11I**) also suggests that daily distance travelled by the cell is not enough for the cell exiting the field of view.

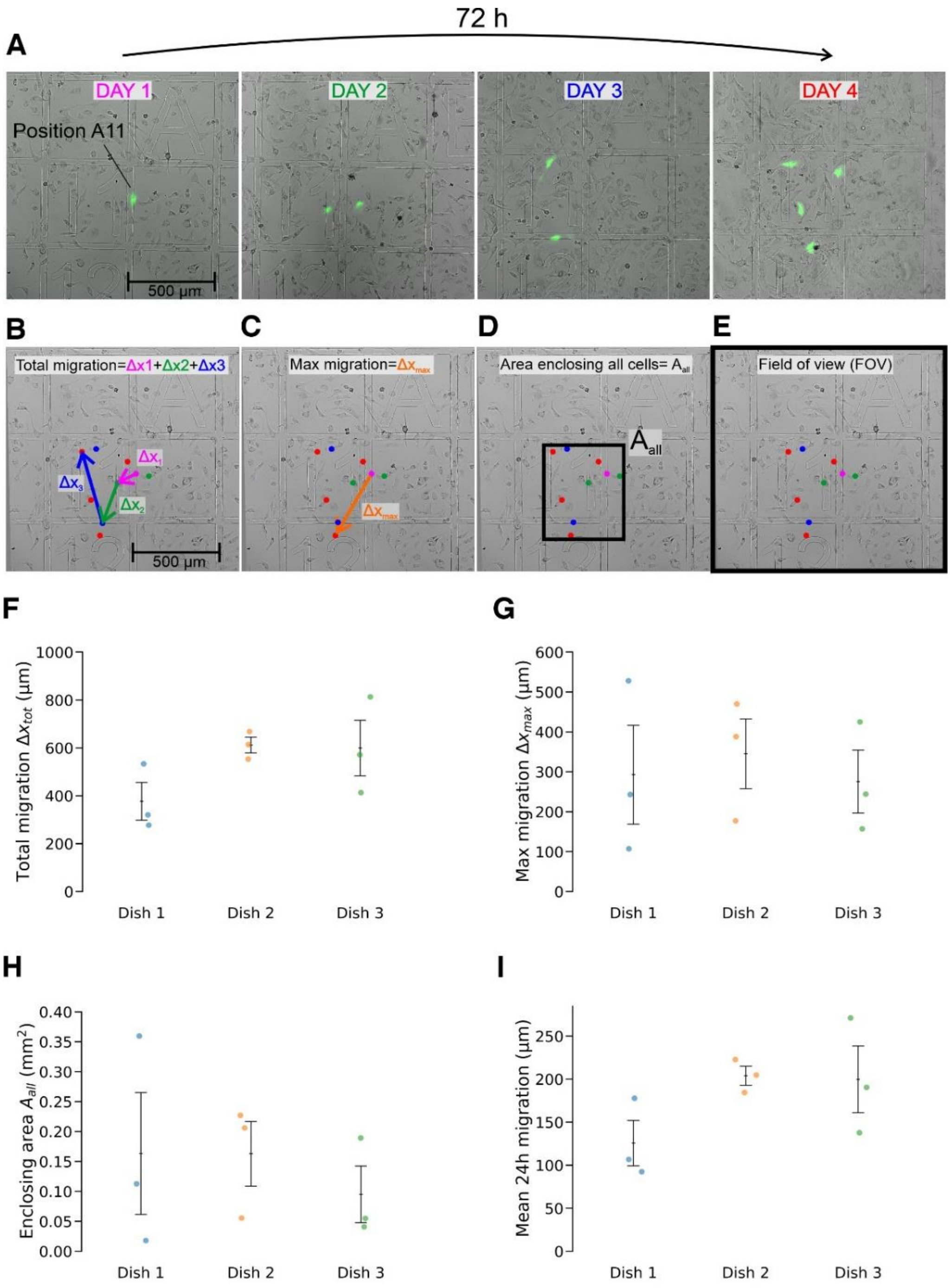

Figure S11: Analysis of cell migration and division over 72 hours. (A) a single GFP-transfected cell is identified on day 1 in a specific location on the grid (A11) and it is tracked over 72 hours (DAY1-DAY4). An image processing routine allowed the quantification of the total cell migration (B), the maximum cell migration over 72 hours (C), and the area enclosing all cells after 72 hours (D). (E) The field of view (FOV) defines the observable area, and it is imposed by the microscope objective and camera (1300 x 1300 µm using 10x magnification objective). The total cell migration (F), the maximum migration over 72 hours (G), the area enclosing all cells after 72 hours (H), and the mean cell migration over 24 hours (I) were measured from 3 cells across 3 different dishes. All parameters suggest that the extent of cell migration does not exceed the dimensions of the field of view. Error bars indicate the standard error of

2.4 Probability of finding the same cell or its progeny after 72 hours

We extracted the data for the average migration distance over 24 hours (**Fig. S12A**) and we established that the dataset follows a normal distribution (Shapiro-Wilk test, p-value=0.61, accept H0 hypothesis of data normally distributed). Next, we estimated the probability density function (**Fig. S12B**) for the distance travelled by the cell over 72 hours by considering the migration speed = 4.21 µm/h) and its standard deviation (std_speed = 2.35 µm/h). Given the time course of the experiment of 72 hours, we calculated the average migration over 72 hours (303 µm) and its standard deviation (169 µm). Given the size of the field of view (FOV = 1300 x 1300 µm) and the cell positioned at its centre (0) on day 1, the maximum distance the cell can migrate before exiting the field of view is 650 µm, assuming migration along a single cartesian axis. Given the probability density function (Fig. SF2.4B), the probability of a cell migrating out of the field of view is equal to 0.02 = 2%. Given the probability to find the same cell within the field of view (1-0.02 = 0.98), the probability of finding the same cell over 72 hours depends on the likelihood that a progeny is generated following biopsy, and the death rate of progeny cells over 72 hours. We calculated the average doubling time (DT) for cells in the data shown in **Fig. S15** (DT = 27h). For ease of calculation, we approximated it to 24 hours, meaning that a cell generates two progeny cells every 24 hours. This means that in the worst-case scenario, a cell will undergo three divisions over 72 hours generating 8 cells. The probability of finding the same cell would be 1/8 = 0.125 = 12.5%.

Under the hypothesis that the biopsied cell survives (survival rate in main manuscript) and considering the average number of progeny cells that die over 72 hours extracted from the data in **Fig. S15** (average number of dead cells DC = 0.73 ~ 1), the total number of progeny cells after 72 hours would be 8-1 ~ 7 cells. This would increase the probability of finding the same cell to 1/7 = 0.142 = 14.2%. Combining the probability of finding the same cell considering progeny generation, progeny death rate and migration, we obtain the probability that the cell biopsied after 72 hours corresponds to the original cell biopsied on day 1 with value corresponding to 0.142 x 0.980 = 0.139 = 13.9%. This probability would increase exponentially at the

decreasing of the number of cell divisions over 72 hours up to 98% in the case the biopsied cell does not divide over the course of the 72 hours, which was a frequent observation as **Fig. S16** shows.

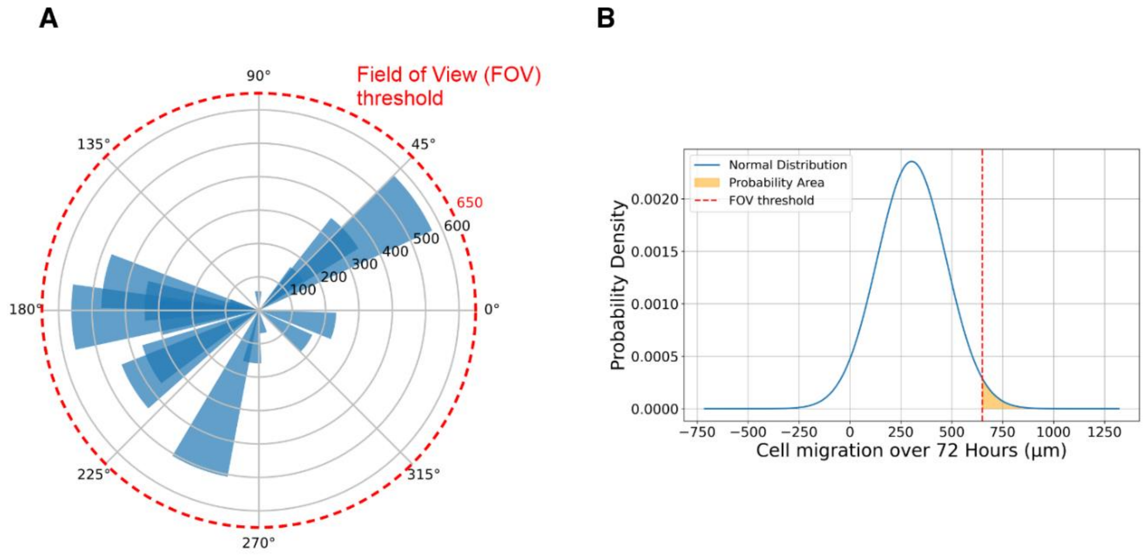

Figure S12: Migration over 72 hours and its probability density function. (A) Rose plot illustrating the migration over 72 hours calculated from the migration speed in relation to the field of view (FOV) size. (B) Probability density function for the migration over 72 hours. The probability of a cell migrating beyond the FOV threshold is equal to 0.02.

2.5 Longitudinal sampling of the same glioblastoma cell

To determine whether the same cell could be sampled at different time points, a mixed culture of $M059K_{GFP}$ and $M059K_{WT}$ was plated on a gridded dish, as described in **Section 2.3**, and an individual $M059K_{GFP}$ was identified near the position H20 on the grid using a 10x magnification objective, ensuring the presence of only one $M059K_{GFP}$ in the field of view. Next, a nanobiopsy was collected resulting in the cell emitting a red fluorescence signal (false yellow color) following nanoinjection of the red fluorophore ATTO 565 (**Fig. S13A**). The dish containing the nanobiopsied cell was brought back to the incubator and the cell was imaged again after 24 hours (day 2, **Fig. S13B**) and 48 hours (day 3, **Fig. S13C**) and was found approximately in the same position (H20) on the grid. After 72 hours (day 4), a longitudinal nanobiopsy was collected resulting in the cell emitting a red fluorescence signal following nanoinjection (**Fig. S13D**).

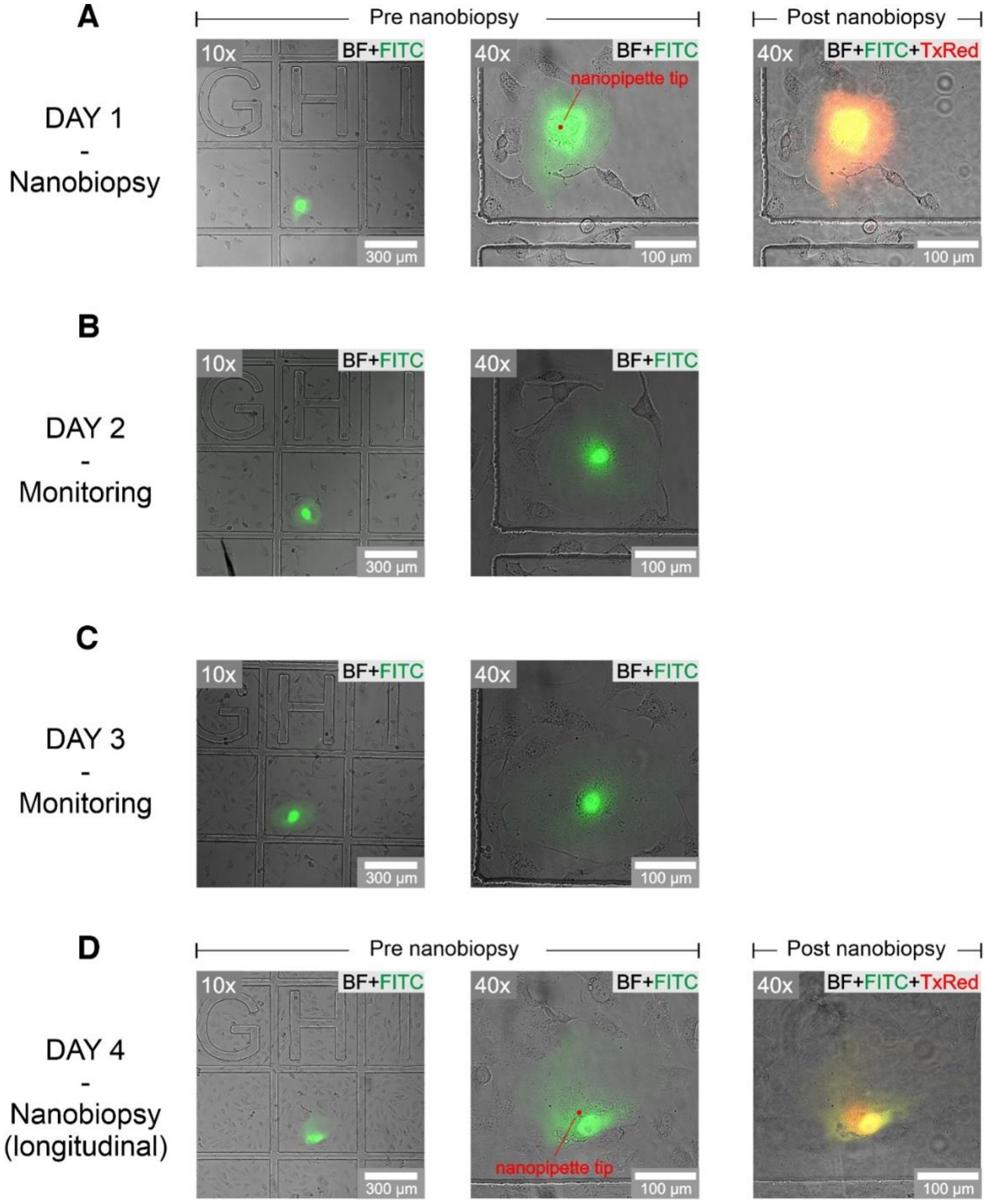

Figure S13: Longitudinal nanobiopsy of the same cell. On day 1 (A), a nanobiopsy was collected from an individual M059K$_{GFP}$ localized in position H20 on the grid resulting in the cell emitting a red fluorescent signal following nanoinjection of a red fluorophore (false yellow color). The cell was imaged again on day 2 (B) and day 3 (C) when it was found around the same position H20. On day 4 (D), a longitudinal nanobiopsy was performed resulting in the cell emitting a red-fluorescent signal following nanoinjection. Micrographs were obtained using the filter set for bright field (BF), fluorescein isothiocyanate (FITC) and texas red (TxRed). The fluorophore injected was ATTO 565.

2.6 Examples of longitudinal nanobiopsies on untreated cells

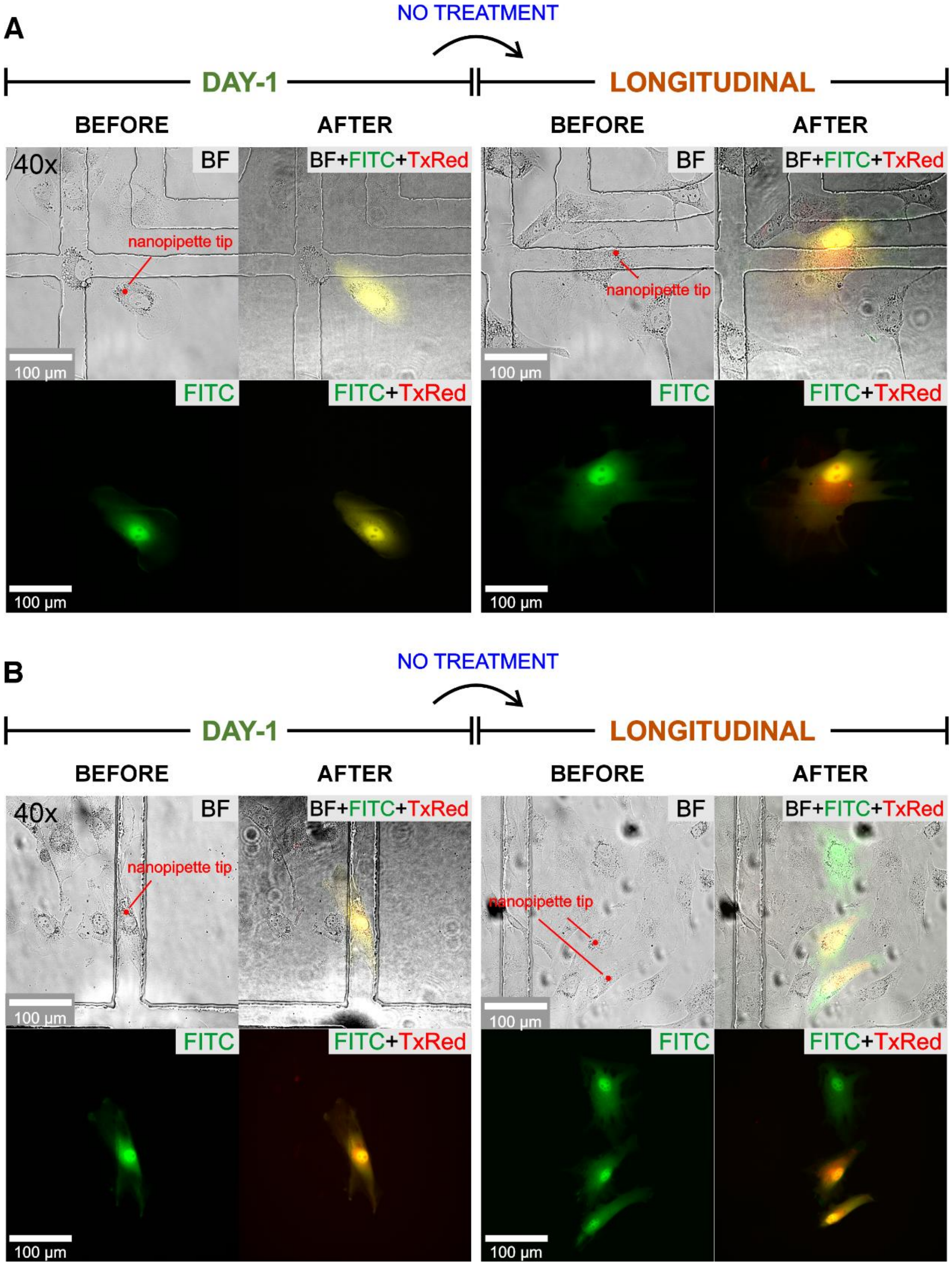

Figure S14: Examples of longitudinal nanobiopsies collected from untreated M059K GBM cells. (A) Optical (BF) and fluorescence (FITC, TxRed) micrographs of an individual $M059K_{GFP}$ cell that is nanobiopsied and nanoinjected on day 1 that is left untreated and nanobiopsied and nanoinjected again on day 4 (longitudinal). (B) Optical and fluorescence micrographs of an individual $M059K_{GFP}$ cell that is nanobiopsied and nanoinjected on day 1, survive over 72 hours and divide, whose progeny is nanobiospied and nanoinjected a second time on day 4.

2.7 Cell survival and division rate over 72 hours for control cells

The same approach as the one described in **Section 2.3** was used to determine the survival and division rate of individual M059K$_{GFP}$ control (not biopsied, untreated) cells monitored over 72 hours. The heatmap in **Figure S15** shows the number of cells present in the same location on the grid after at 0,24, 48 and 72 hours obtained by monitoring 41 cells over 72 hours. 26/41 cells survived over the 72 hours with a survival rate of ~63%. The cell division rate varied across different samples with an average of 4 cells present after 72 hours (2 division cycles). However, this number is merely approximative as the number of cells after 72 hours was variable with some cases characterised by 9 cells present in the same location on the grid (>3 division cycles).

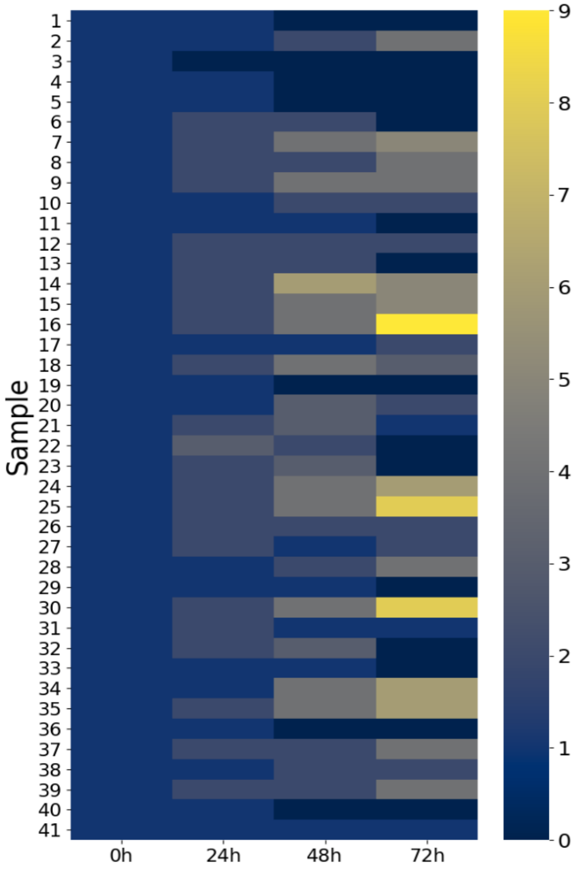

Figure S15: Heatmap showing cell division over 72 hours for control (not biopsied, untreated) cells. The color indicates the number of cells found in the same location on each day (columns) for each sample (row). The total number of cells tracked (rows) was 41. The 0h column shows uniform color due to one individual M059K$_{GFP}$ cell being identified, thus all values are equal to 1. When the initial cell dies, a dark blue color indicates 0 cells.

2.8 Cell population 72 hours post nanobiopsy and control group

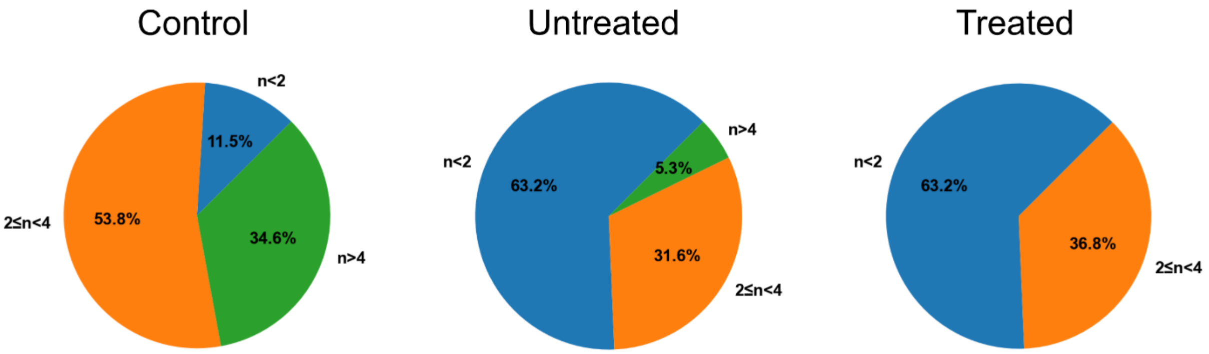

Figure S16: Cell population post nanobiopsy. Pie charts showing the percentage of cases where <2, ≤2 and >4, and >4 cells were present 72 hours after plating in the control group, and 72 hours following nanobiopsy for the untreated and treated samples. The total number of samples was 39, 19, and 19 for the control, untreated and treated group, respectively. The higher percentage of cells in the n<2 group for both the untreated and treated samples suggest a decreased division rate in the case of biopsied cells (Chi-squared test, Untreated vs. Control p-value = 0.0008, Treated vs Control p-value = 0.0003).

2.9 Effects of nanobiopsy on cell mobility

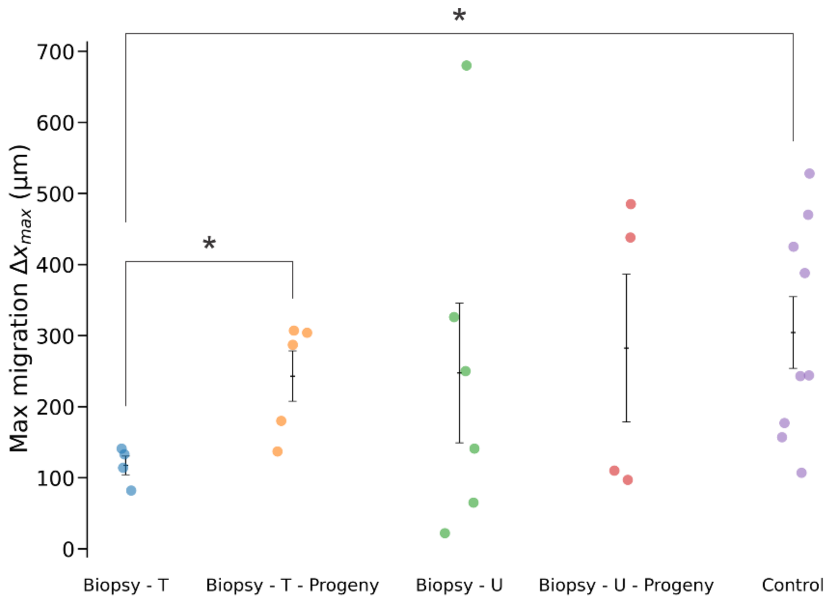

Figure S17: Effects of nanobiopsy on cell mobility. The graph shows the maximum migration Δx_{max} for cells that were biopsied, treated that did not divide (Biopsy-T) and that divided (Biopsy-T-Progeny) over the 72 hours post-biopsy, and for cells that were biopsied, left untreated, that did not divide (Biopsy-U) and that divided (Biopsy-U-Progeny) over the 72 hours post-biopsy compared to the control group (Control). A significant difference was observed between Biopsy-T and Control (standard t-test, p=0.035) and between Biopsy-T and Biopsy-T-Progeny (standard t-test, p=0.020). Error bars indicate the standard error of the mean.

2.10 Quality control of multiplexed single-cell libraries

Two multiplexed libraries were generated for the whole-cell lysate (positive control) and nanobiopsy samples, respectively. A tape station was used to determine the fragment size of both libraries.

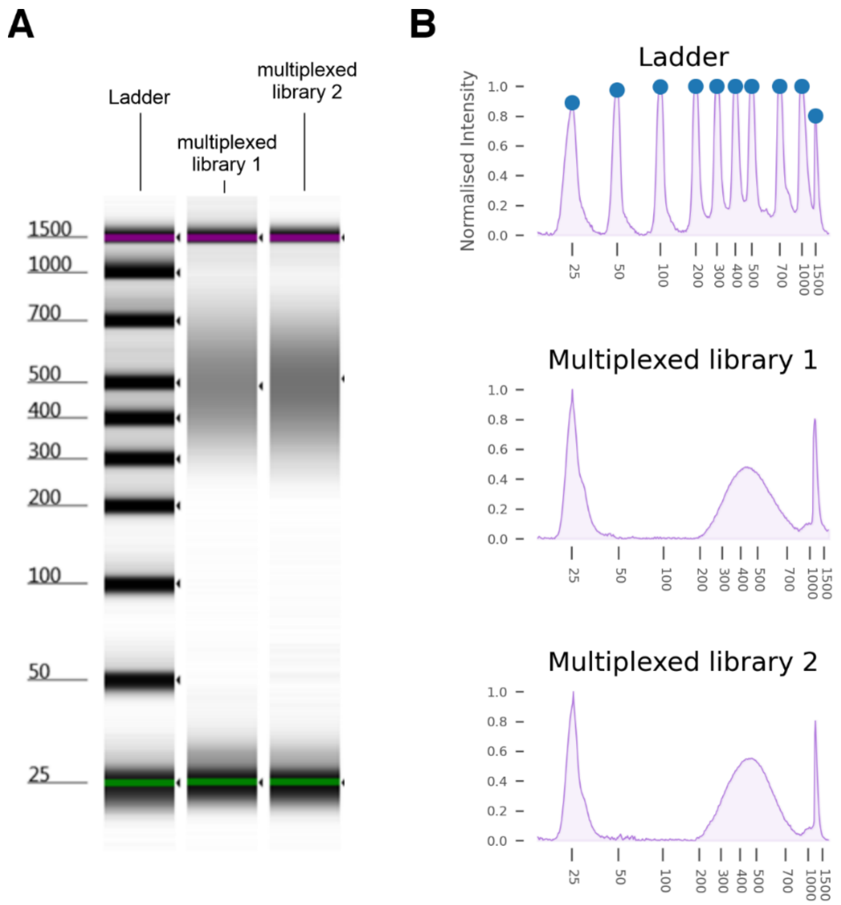

Figure S18: Quality control of multiplexed single-cell libraries. (A) Tape station traces showing the calibration ladder, the multiplexed library 1 containing the whole-cell lysate samples and the multiplexed library 2 containing all the nanobiopsy samples. (B) densitometry traces extracted from the tape station images showing the fragment size distribution of both multiplexed libraries.

3: Sequencing data analysis

3.1 Quality metrics of unfiltered data

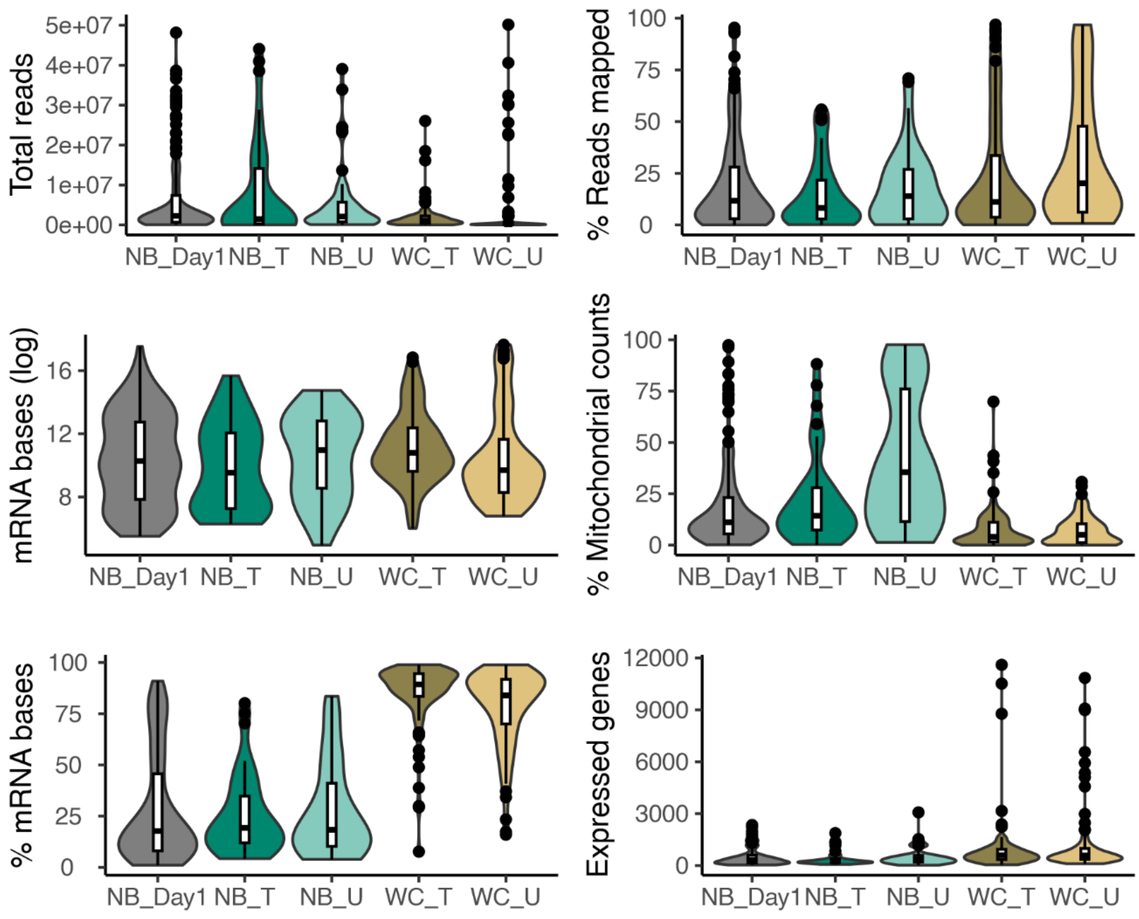

Figure S19: Main sequencing metrics before data filtering. The groups correspond to day-1 nanobiopsy (NB_Day1) longitudinal nanobiopsy of treated (NB_T) and untreated (NB_U), and whole-cell lysate treated (WC_T) and untreated (WC_U) samples.

3.2 Effect of different volume amounts extracted on the sequencing data

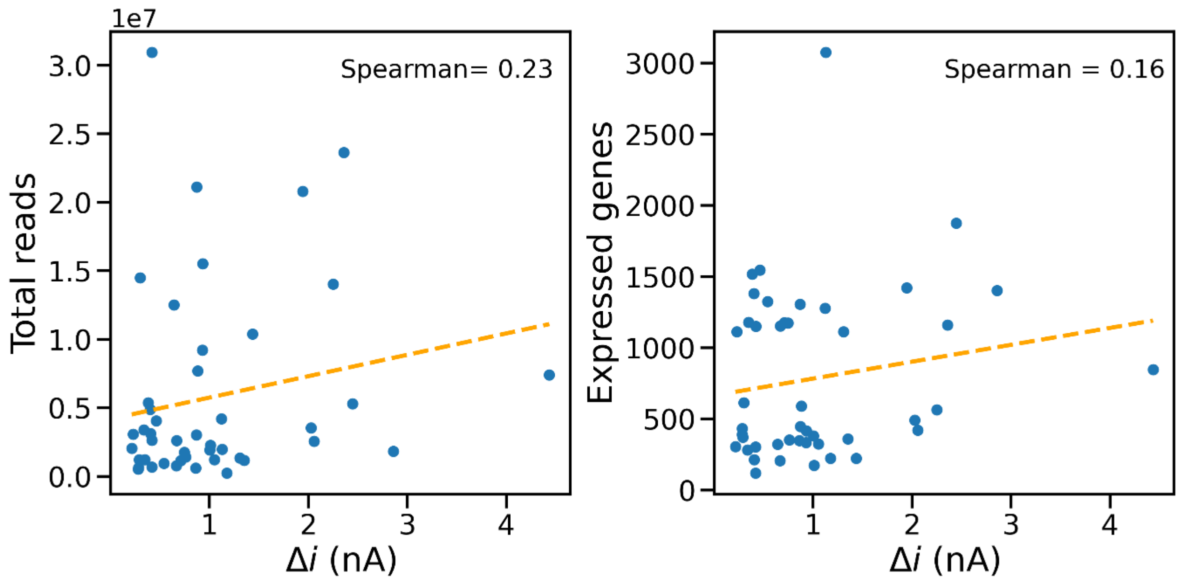

Figure S20: Correlation between ion current increase and sequencing metrics. Correlation between the increase in ion current Δi_{org} and the total number of reads (left) and the number of expressed genes (right) calculated for the nanobiopsy samples that passed filtering for which the ion current traces were available for analysis (n = 45).

3.3 Contamination control and effect of electrowetting

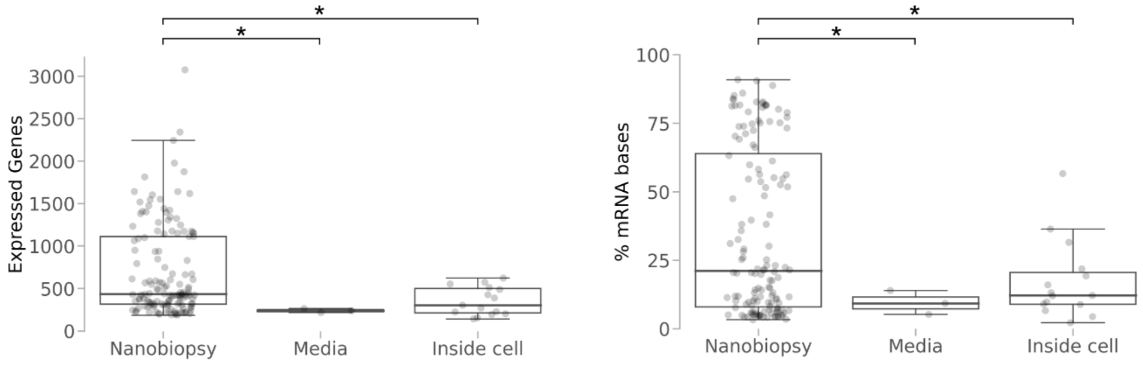

Figure S21: Contamination due to the culture medium and effect of electrowetting on the extracted material. The nanobiopsy samples (Nanobiopsy) show a significantly higher number of expressed genes and % mRNA bases when compared to the control samples obtained by immersing the nanopipette in the culture medium with cells (Media) and the control samples obtained by penetrating the cellular membrane without applying electrowetting (Inside cell). Expressed genes, Welch's t-test, p-value = 4.47e-16 (Nanobiopsy – Media), p-value = 8.12e-7 (Nanobiopsy – Inside cell), % mRNA bases, Welch's t-test, p-value = 1.22e-4 (Nanobiopsy – Media), p-value = 6.87e-4 (Nanobiopsy – Inside cell).

3.4 Principal component analysis visualization of longitudinal nanobiopsy data.

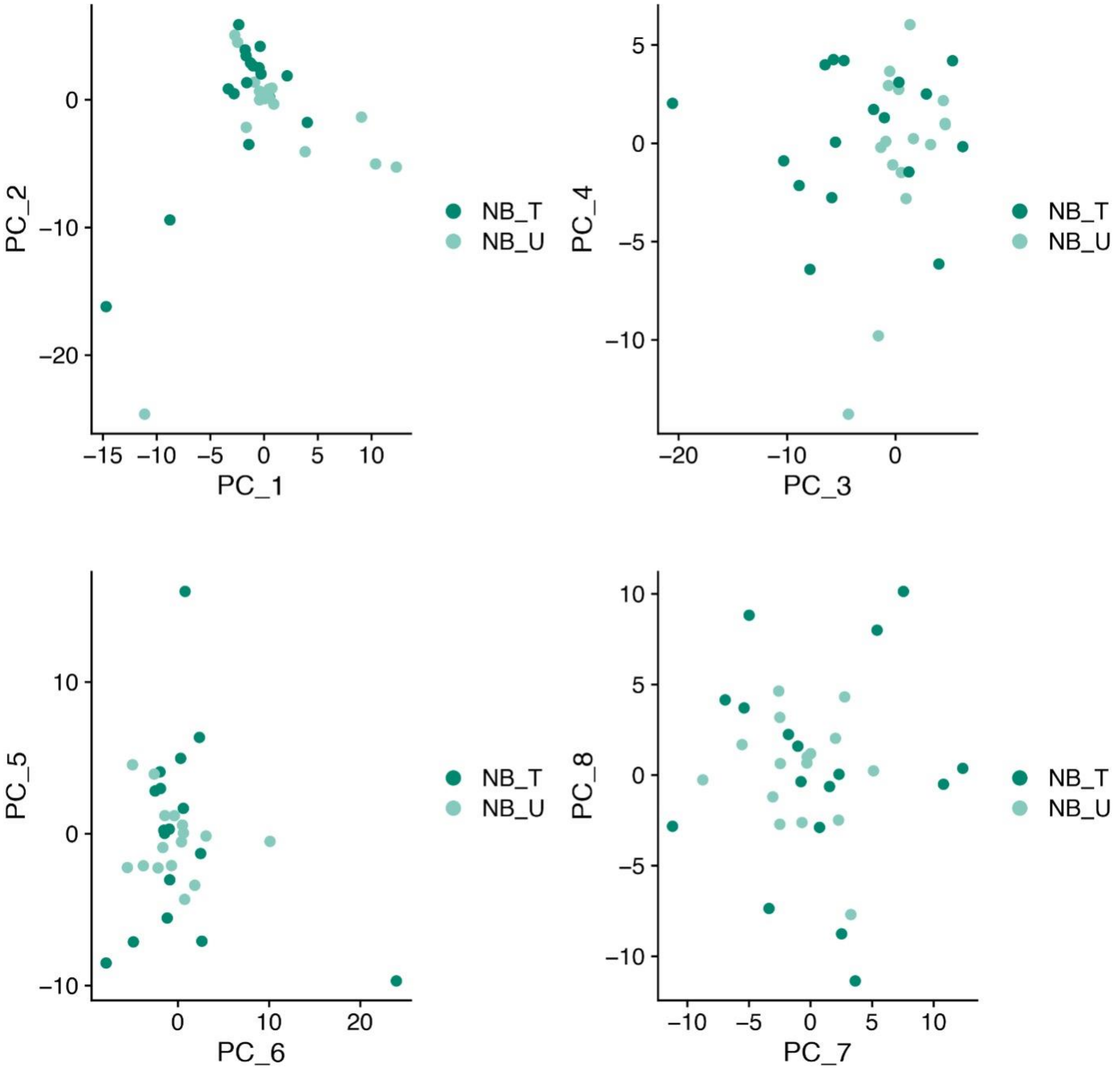

Figure S22: Data visualization along principal components. Visualization of longitudinal nanobiopsy data of treated (NB_T) and untreated (NB_U) samples along the first eight principal components.

3.5 Visualization of lack of bias introduced by the metrics on UMAP

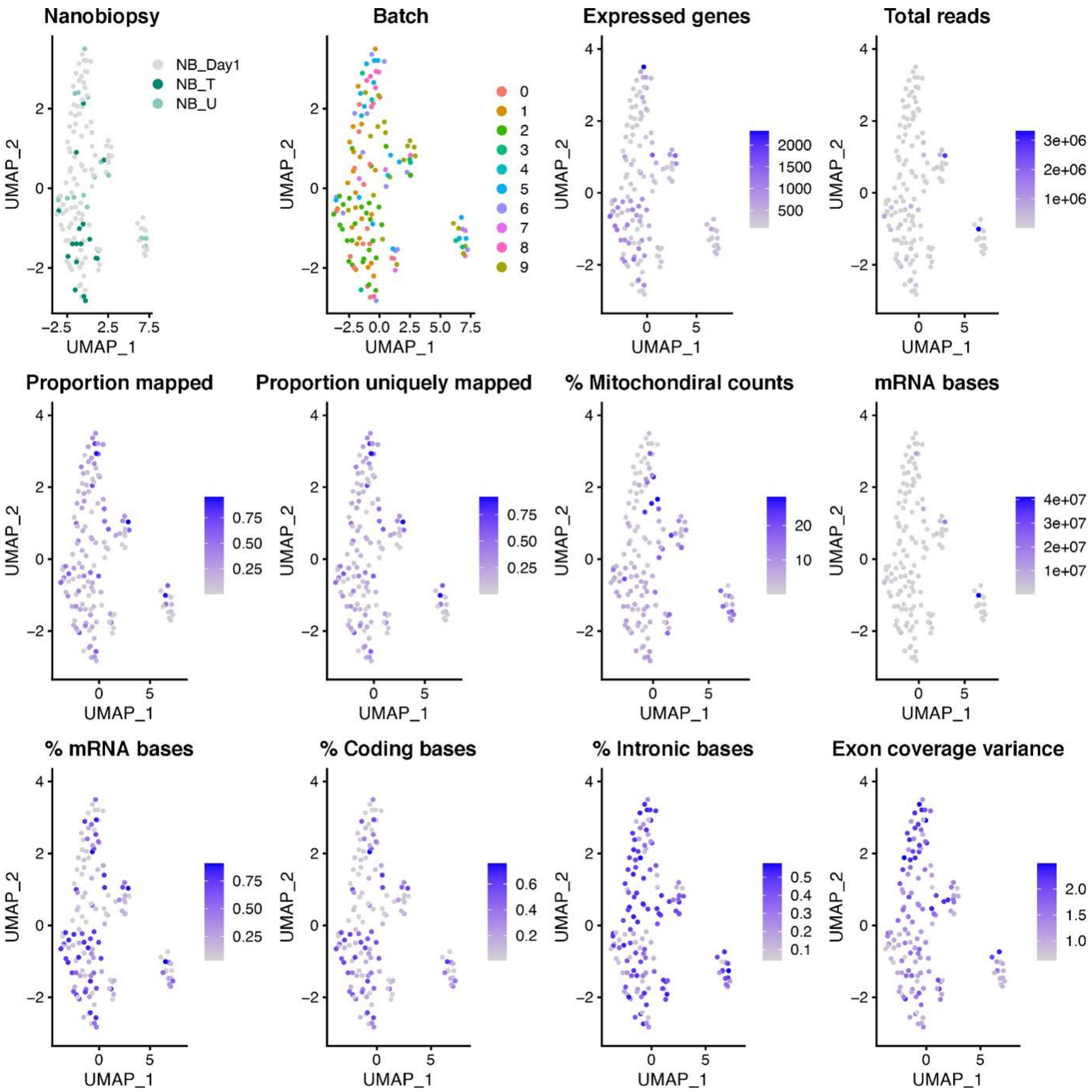

Figure S23: Visualization of technical bias due to batch and sequencing metrics in the UMAP plot.

3.6 Change in the proneural and mesenchymal score of treated and untreated longitudinal nanobiopsy samples

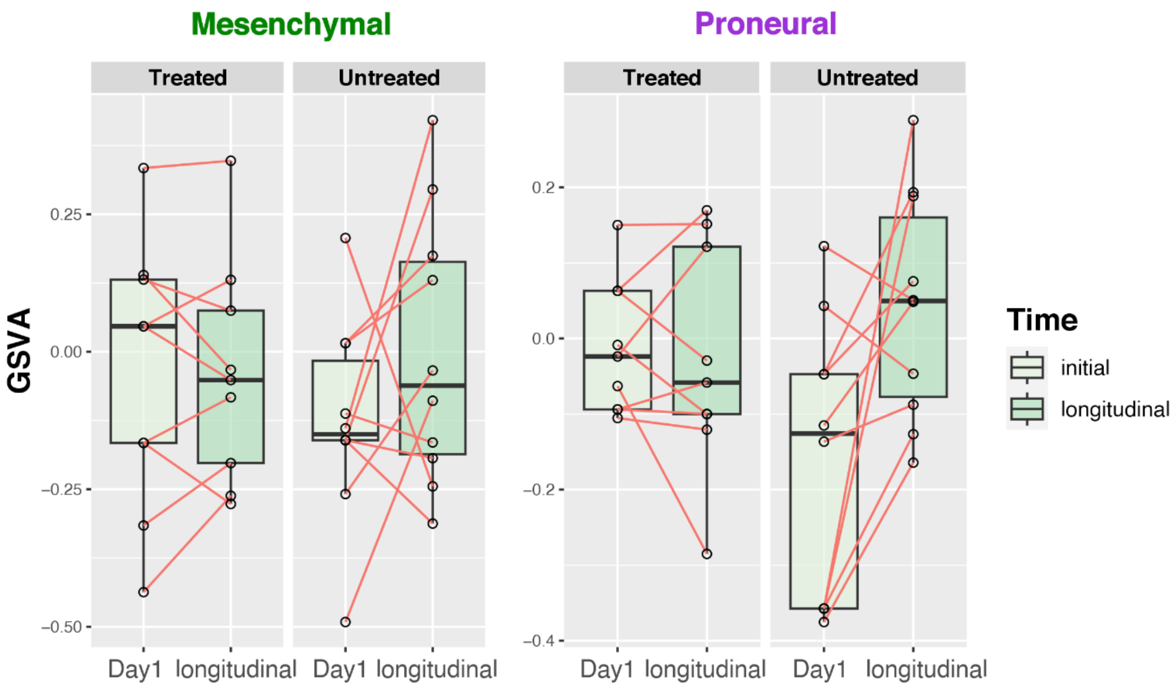

Figure S24: Change in PN and MES score of treated and untreated nanobiopsy samples. Boxplots showing the individual GSVA score for the mesenchymal (green) and proneural (purple) subtype of paired day1 and longitudinal treated and untreated nanobiopsy samples.

3.7 Distributions of phenotypes in whole-cell and nanobiopsy samples

Whole-cells

	Treated	Untreated
**Pro**	43	39
**Mes**	24	30

Nanobiopsies

	Treated	Untreated
**Pro**	13	10
**Mes**	4	6
